# A spatial map of human macrophage niches reveals context-dependent macrophage functions in colon and breast cancer

**DOI:** 10.21203/rs.3.rs-2393443/v1

**Published:** 2023-01-10

**Authors:** Magdalena Matusiak, John W. Hickey, Bogdan Luca, Guolan Lu, Lukasz Kidziński, Shirley Zhu, Deana Rae Crystal Colburg, Darci J. Phillips, Sky W. Brubaker, Gregory W. Charville, Jeanne Shen, Garry P. Nolan, Aaron M. Newman, Robert B. West, Matt van de Rijn

**Affiliations:** 1 Department of Pathology, Stanford University, Stanford, California, USA.; 2 Stanford Center for Biomedical Informatics Research, Department of Medicine, Stanford University, Stanford, California, USA.; 3 Department of Biomedical Data Science, Stanford University, Stanford, California, USA; 4 Department of Bioengineering, Stanford University, Stanford, CA, USA; 5 Department of Microbiology and Immunology, Stanford University, Stanford, CA, USA; 6 Institute for Stem Cell Biology and Regenerative Medicine, Stanford University, Stanford, California, USA.; 7 Stanford Cancer Institute, Stanford University, Stanford, California, USA.

## Abstract

Tumor-associated macrophages (TAMs) display heterogeneous phenotypes. Yet the exact tissue cues that shape macrophage functional diversity are incompletely understood. Here we discriminate, spatially resolve and reveal the function of five distinct macrophage niches within malignant and benign breast and colon tissue. We found that SPP1 TAMs reside in hypoxic and necrotic tumor regions, and a novel subset of FOLR2 tissue resident macrophages (TRMs) supports the plasma cell tissue niche. We discover that IL4I1 macrophages populate niches with high cell turnover where they phagocytose dying cells. Significantly, IL4I1 TAMs abundance correlates with anti-PD1 treatment response in breast cancer. Furthermore, NLRP3 inflammasome activation in NLRP3 TAMs correlates with neutrophil infiltration in the tumors and is associated with poor outcome in breast cancer patients. This suggests the NLRP3 inflammasome as a novel cancer immunetherapy target. Our work uncovers context-dependent roles of macrophage subsets, and suggests novel predictive markers and macrophage subset-specific therapy targets.

## Introduction

TAM infiltration, as measured by CD68 immunohistochemistry (IHC), predicts poor patient outcomes for most tumor types (Fridman et al. 2017), indicating that macrophages play a critical role in the tumor microenvironment (TME). As a result, TAMs were surmised to be a promising cancer therapy target. However, TAM targeting therapeutic efforts have shown minimal single-agent efficacy against solid tumors, including CSF1 pathway blockade (Papadopoulos et al. 2017; Ries et al. 2014). This may be in part because such therapies treat macrophages as a single entity and aim to repress macrophage biology as a whole. Clearly, a better understanding of the molecular and functional diversity of TAMs is needed to facilitate rational macrophage targeting in cancer and predict clinical outcomes.

Previous studies revealed transcriptional macrophage heterogeneity in human cancer (Azizi et al. 2018; Zhang et al. 2020; Mulder et al. 2021), but it was not clear which of the identified single-cell clusters corresponded to functionally distinct subsets. We and others used immunostaining to show that macrophage markers including MARCO, APOE, CCR2, TREM2, and FOLR2 are restricted to spatially discrete macrophage populations (La Fleur et al. 2018; Luca et al. 2021; Nalio Ramos et al. 2022) and demonstrated their differential spatial co-enrichment with distinct T cell subtypes (Luca et al. 2021; Nalio Ramos et al. 2022). However, these immunostaining studies were limited to examining one or two macrophage and T cell populations at a time in a single organ system. Unbiased and highly multiplexed profiling across all tissue cell types and different organ systems is needed to fully dissect macrophage spatial tissue organization and cell-cell interactions that shape macrophage functions in the TME.

Here we link single-cell RNA sequencing (scRNA Seq) data with multiplex immunofluorescence (mIF) to discriminate five discrete macrophage populations (LYVE1 TRM, FOLR2 TRMs, IL4I1 TAMs, NLRP3 TAMs and SPP1 TAMs) in human breast cancer (BC), colorectal cancer (CRC), and their benign counterparts. The uniqueness of our approach is based on 1) profiling of all macrophage populations in the TME using subtype-specific protein markers, and 2) unbiased spatial profiling that allows us to discover novel spatial associations between macrophage subtypes and almost all other cell types in the TME. We show that the different macrophage populations occupy spatially distinct niches characterized by unique cellular compositions and discrete functional properties, demonstrating they correspond to biologically distinct populations. We found SPP1 TAMs are associated with hypoxia and tumor necrosis, and a novel subset of FOLR2 TRMs is enriched in the plasma cell niche. We show that IL4I1 macrophages are actively phagocytosing, are likely targets of anti-CD47 and anti-PD-L1 immunotherapies, and correlate with anti-PD1 treatment response. Furthermore, we demonstrated that NLRP3 TAMs activate the inflammasome in breast cancer (BC), colorectal cancer (CRC), and Crohn’s Disease (CD). NLRP3 inflammasome activation is spatially associated with neutrophil infiltration, suggesting that inflammasome activation contributes to neutrophil tissue accumulation in cancer. Finally, NLRP3 TAMs and neutrophil niche abundance correlate with outcomes in BC patients and thus suggest NLRP3 inflammasome blockade as a novel therapeutic target in cancer and CD. This work conceptualizes the macrophage niche as a fundamental and conserved functional tissue building block, demonstrates strategies to identify and further study distinct macrophage populations *in situ* in human clinical specimens, and identifies new candidate predictive markers and macrophage-targeted cancer therapy targets.

## Results

### Experimental approach

**Fig 1.**
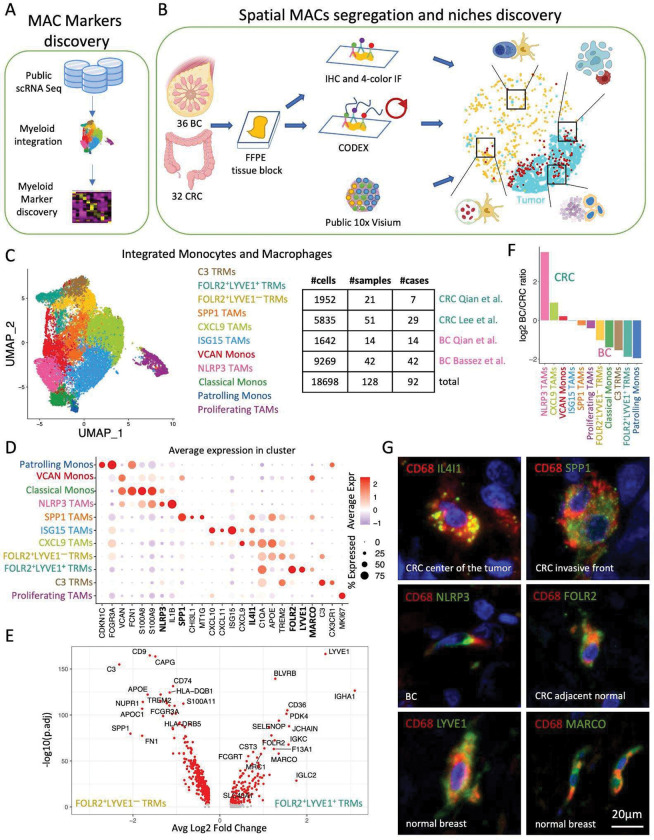
ScRNA Seq reveals differences in spatial enrichment of myeloid markers. (**A** and **B**) Flow charts of experimental design. (**C**) UMAP projection of monocyte and macrophage scRNA transcriptomes from 4 studies colored by annotated populations (*left*) and a breakdown of cells, samples and patient numbers by study (*right*). (**D**) Dotplot of average marker gene expression per scRNA myeloid population. Highlighted in bold are 6 markers for which FFPE-compatible antibodies were identified. (**E**) Volcano plot shows top differentially expressed genes between FOLR2^+^, LYVE1^−^ and FOLR2^+^, LYVE1^+^ TRMs. (**F**) Barplot of the ratio of log2 average fractional scRNA myeloid population enrichment between CRC and BC in tumor samples with more than 35 monocytes and macrophages detected. (**G**) Immunofluorescence images show overlap of the established FFPE antibodies and CD68, confirming their reactivity with macrophages.

**Fig 2.**
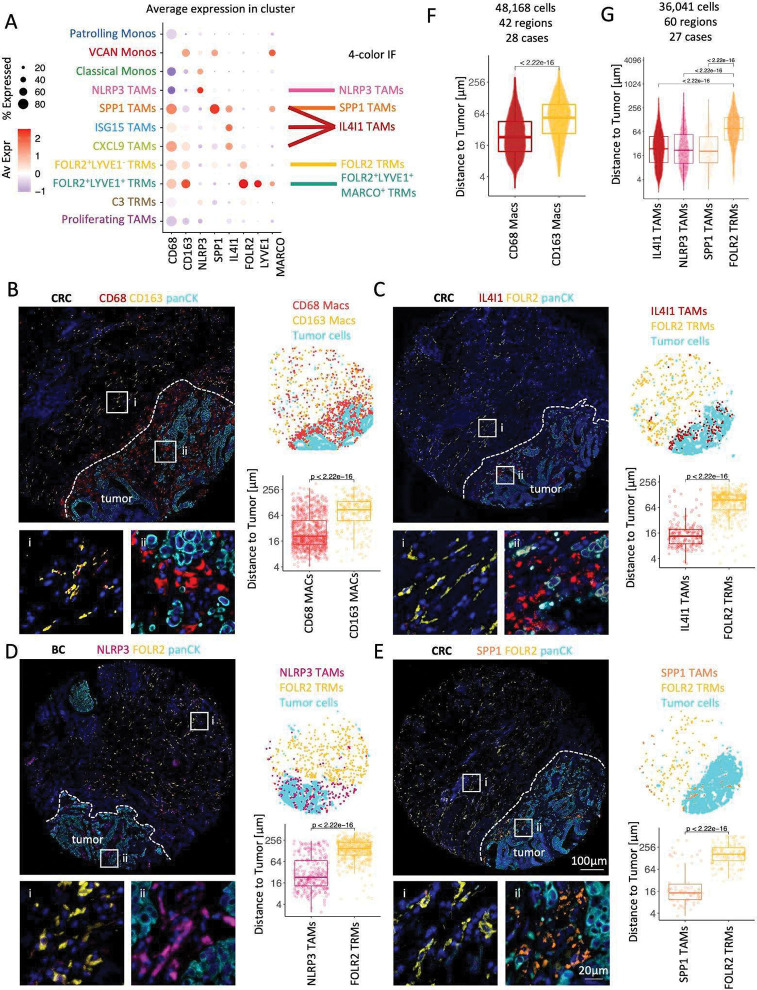
FOLR2, IL4I1, NLRP3, and SPP1 mark spatially distinct macrophage niches in the TME. (**A**) Dotplot shows average macrophage marker expression in scRNA macrophage populations and indicates which scRNA macrophage populations are detectable in 4-color IF staining by anti-NLRP3, -SPP1, -IL4I1, -FOLR2, and a combination of anti-FOLR2, -LYVE1 and -MARCO antibodies. (**B-E**) *Left:* CODEX image (**B**) or Immunofluorescence (IF) images (**C,D,E**) show the distribution of CD68 and CD163 (**B**), or FOLR2 and IL4I1 (**C**), NLRP3 (**D**), SPP1 (**E**) protein expression in representative cases of CRC (**B**,**C,E**) and BC (**D**). PanCK marks tumor cells. Close-up images on the bottom correspond to boxed regions on the top. *Top right:* Scatterplots show the distribution of CD68 Macs, CD163 Macs, FOLR2 TRMs, IL4I1 TAMs, NLRP3 TAMs, SPP1 TAMs corresponding to IF images on the left. *Bottom right:* Boxplots show the distance quantification of each macrophage to the closest tumor cell corresponding cells identified on IF images on the left. Pairwise comparisons were determined using a two-sided Wilcoxon rank-sum test on 1092 (**B**) 580 (**C**), 739 (**D**), and 203 (**E**) cells. (**F**) Distance (μm) of CD68 and CD63 macrophages to the closest tumor cell. (**G**) Distance (μm) of IL4I1 TAMs, NLRP3 TAMs, SPP1 TAMs, FOLR2 TAMs to the closest tumor cell. (**F,G**) Cells were identified on CODEX images, *P values* were calculated with a linear mixed-effect model with Bonferroni’s corrections for multiple comparisons.

**Fig 3.**
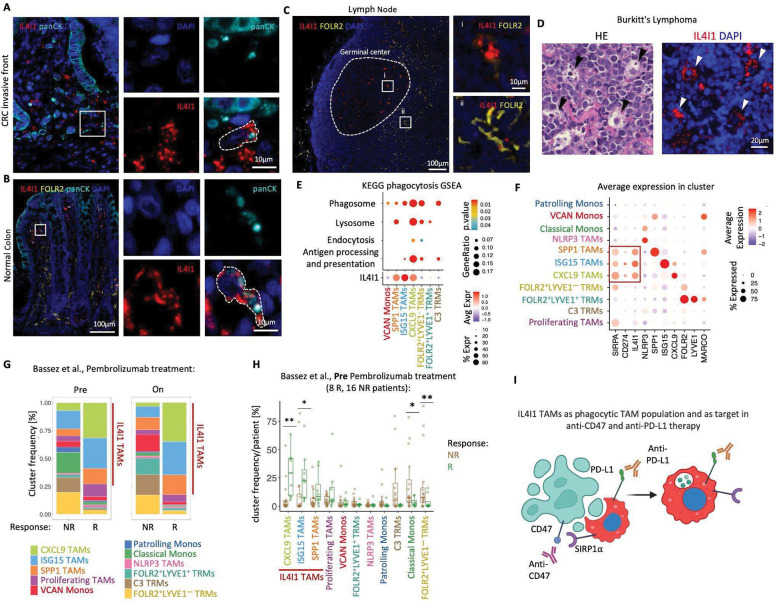
IL4I1 marks phagocytosing macrophages. (**A**) IF images of invasive front of CRC stained with IL4I1, FOLR2, panCK, and DAPI show the presence of panCK^+^ material within IL4I1 macrophages. (**B**) Same as (**A**) but normal colon mucosa. (**C**) IF images of normal Lymph Node stained with IL4I1, FOLR2, and DAPI. (i) is a close-up image of a germinal center tingible body macrophage (TBM), (ii) is a close-up image of interfollicular FOLR2 TRMs (**A-C**) Close-up images on the right correspond to the boxed region on the left. (**D**) Images of TBMs in Burkitt’s lymphoma stained with *left*: H&E and *right*: IL4I1 and DAPI. (**E**) *Top:* KEGG pathways enrichment analysis of phagocytosis-related pathways across scRNA macrophage populations. Populations with no significantly enriched pathways were omitted. *Bottom:* average IL4I1 gene expression across scRNA macrophage populations with enriched phagocytosis-related gene sets. (**F**) Dotplot shows average gene expression in scRNA macrophage populations. (**G**) Barplots show frequency of scRNA monocyte and macrophage clusters in dataset from Bassez et al., stratified by response to pembrolizumab and time of sample collection. (**H**) Boxplots show frequency of scRNA monocyte and macrophage clusters pre pembrolizumab treatment from Bassez et al. (**I**) Schematic illustrating IL4L4I1 TAM association with cell death and efferocytosis and highlighting IL4I1 TAMs as potential anti-CD47 (indirect as IL4I1 TAMs express CD47 ligand- SIRP1α) and-PD-L1 (direct) therapy targets.

**Fig 4.**
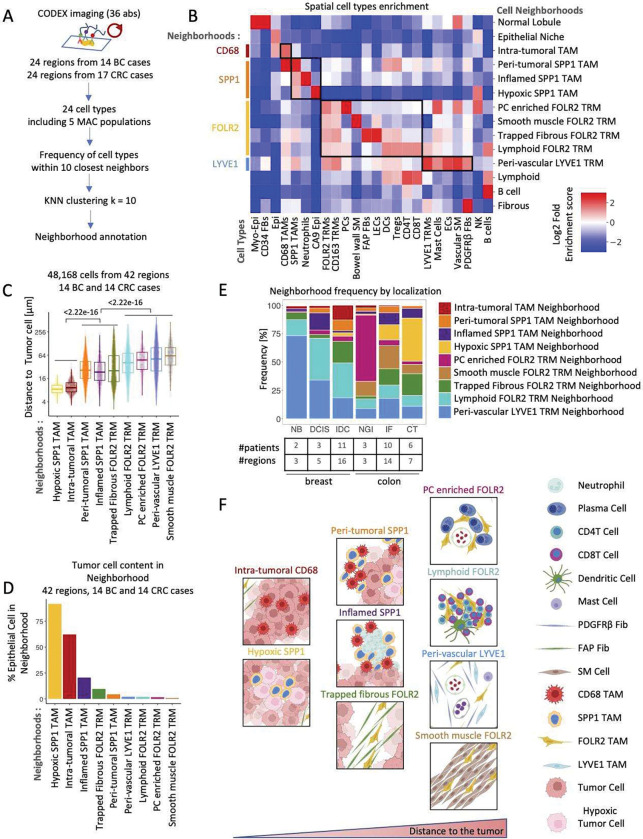
CODEX reveals spatial cellular interactions in macrophage niches within colon and breast cancer tissues. **(A)** Schematic shows CODEX imaging and cellular neighborhood analysis workflow. (**B**) Heatmap shows CODEX cell types (x axis) enrichment (color) in the identified cellular neighborhoods (y axis). (**C**) Boxplot shows distance (μm) to the closest tumor cell for every macrophage identified by CODEX labeled by the neighborhood it belongs to. (**D**) Barplot shows a percentage of the epithelial cells occupied in each CODEX macrophage neighborhood. (**E**) Barplot presents the frequency of CODEX macrophage neighborhoods grouped by anatomical location. NB-normal breast, DCIS-ductal carcinoma in situ breast, IDC-invasive ductal carcinoma breast, NGI-normal GI tract, IF invasive front CRC, CT-center of tumor CRC. (**F**) Schematic shows cellular macrophage neighborhood organization and closeness to the tumor.

**Fig 5.**
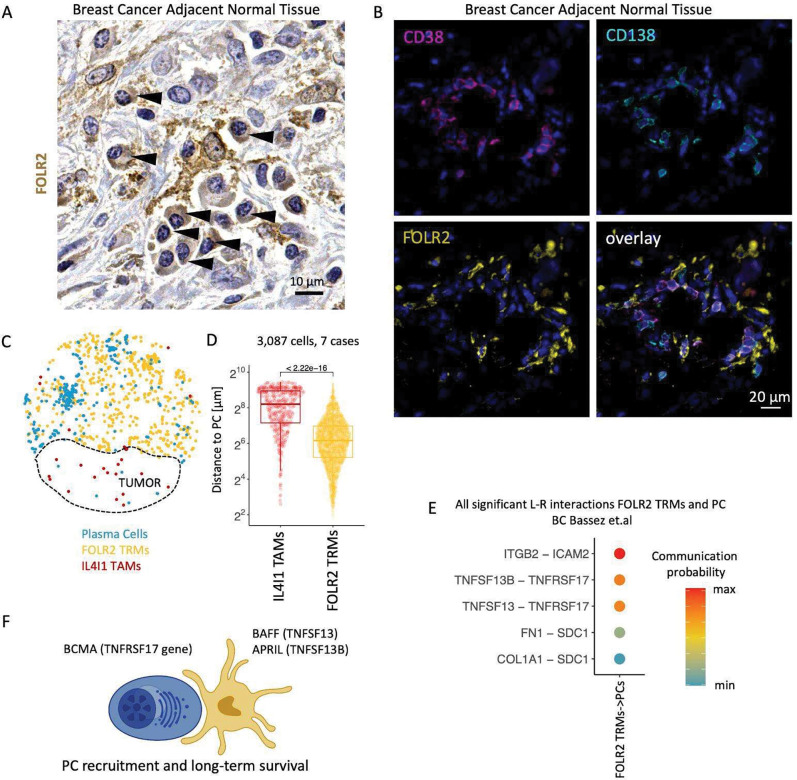
FOLR2 TRMs spatially colocalize with plasma cells and may maintain long-lived plasma cell tissue niche. (**A**) Immunohistochemical image shows FOLR2 TRMs surrounded by plasma cells indicated with black arrows. (**B**) IF images show spatial cell-cell interaction between PCs marked by co-expression of CD38 and CD138 and FOLR2 TAMs marked by FOLR2 located in normal tissue adjacent to BC. Scale bar of 20 μm is identical for all images. (**C**) Scatterplots show the distribution of FOLR2 TRMs, IL4I1 TAMs, and PC identified by CD138 staining in BC TME. (**D**) Boxplot shows distance quantification of each FOLR2 TRMs, IL4I1 TAMs to the closest tumor cell measured across 7 1.5 mm^2^ tissue regions of BC and CRC. *P* value calculated with a linear mixed-effect model. (**E**) Dotplot shows communication probability between all significant Ligand and Receptor interactions between FOLR2 TRMs and PCs in BC scRNA Seq dataset of Basses et. al. (**F**) Schematic illustrating possible FOLR2 TRMs interaction with PCs.

**Fig 6.**
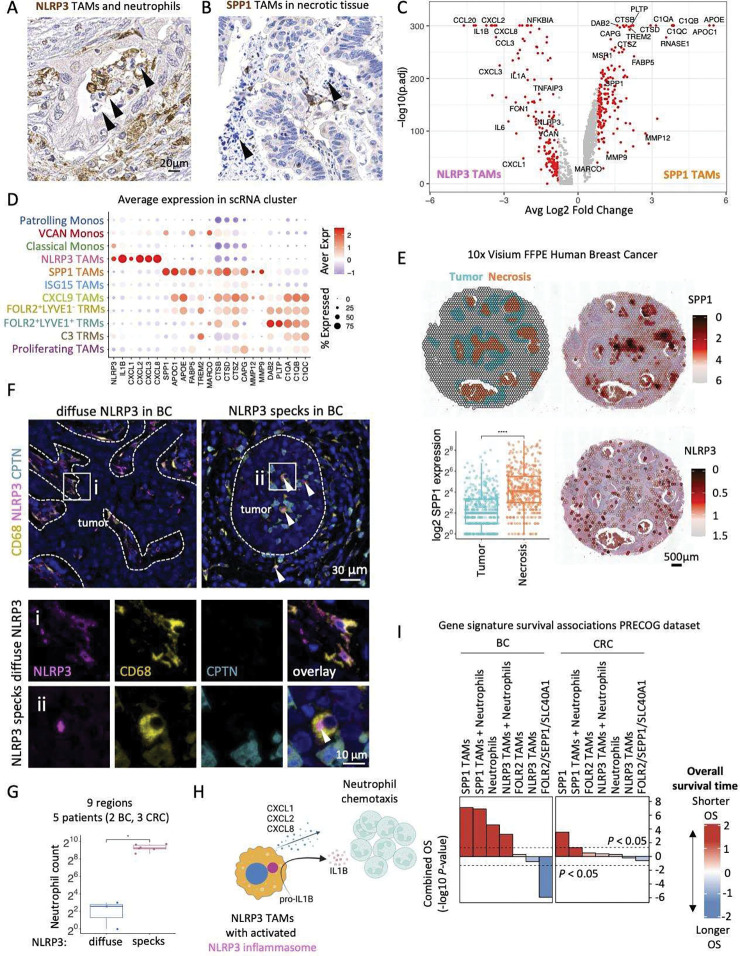
SPP1 TAMs seed hypoxic and necrotic tumor areas and NLRP3 TAMs activate NLRP3 inflammasome in the TME. (**A**) Immunohistochemical image shows NLRP3 TRMs surrounded by neutrophils (arrowheads). (**B**) Immunohistochemical image shows SPP1 TRMs surrounded by karyorrhectic debris in necrotic material (arrowheads). (**C**) Volcano plot shows differential gene expression between scRNA transcriptomes of SPP1 TAMs and NLRP3 TAMs. (**D**) Dotplot of average expression of genes associated with neutrophil chemoattraction, lipid metabolism and phagocytosis across scRNA macrophage populations. (**E**) Dotplot shows the annotation of Tumor (green) and Necrotic (brown) areas (*top left*) and normalized expression of SPP1 (*top right*) and NLRP3 (*bottom right*) on the 10x Visium FFPE Human Breast Cancer sample, and barplot shows normalized log2 SPP1 expression in Tumor and Necrosis regions (*bottom left*). (**F**) Immunofluorescence (IF) shows a representative BC region stained with NLRP3, CD68, Calprotectin (CPTN) and DAPI. Scale bar of 10 μm is identical for all close-up images. (**G**) Quantification of the number of neutrophils present on 9 BC 1.5 mm^2^ tissue regions stratified by whether they contained diffuse NLRP3 (3 regions) or NLRP3 specks (6 regions). *P* value was computed using a two-sided Wilcoxon’s rank-sum test. (**H**) Schematic of a possible mechanism through which NLRP3 TAMs can contribute to the recruitment of neutrophils in the TME. (**I**) Survival associations of single gene or macrophage niche signatures stratified by tumor type.

This work aimed to reveal the cellular composition and spatial tissue distribution of functionally distinct human macrophage niches in the TME. We chose to focus on BC and CRC because CD68 infiltration predicts outcome in BC and CRC patients (Fridman et al. 2017; Beck et al. 2009). We used four public scRNA Seq datasets of CRC and BC (H.-O. Lee et al. 2020; Qian et al. 2020; Bassez et al. 2021) to discover markers of distinct macrophage subtypes (Fig 1A, results in Fig1) and established a panel of 6 antibodies that are compatible with formalin-fixed, paraffin-embedded (FFPE) tissue. These antibodies recognize macrophage markers and identify five discrete macrophage populations *in situ*. We subsequently used whole section IHC, 4-color immunofluorescence (IF), and 36-antibody CODEX assays on Tissue Microarrays (TMAs) to discover distinct spatial macrophage niches and the possible functions these spatially-resolved TAM subsets fulfill in the TME and as TRM in normal tissue (Fig 1B, results in Fig 2–6).

### ScRNA Seq suggests differences in spatial enrichment of myeloid markers.

To find markers for different macrophage populations, we integrated, clustered, and compared scRNA monocyte and macrophage transcriptomes from 18,698 cells from 128 samples derived from 92 patients across four published studies of BC and CRC (Fig 1A, C, S1A). We defined 11 transcriptional clusters marked by differential enrichment of genes (Fig 1C–D). We selected a clustering resolution that separated known myeloid subtypes as follows: TRMs (*LYVE1*^+^) form TAMs (*TREM2*^+^*APOE*^+^), and Patrolling (*CDKN1C*^+^*FCGR3A*^+^) from Classical monocytes (*VCAN*^+^*S100A8*^+^*S100A9*^+^). We differentiated three monocyte, five TAM, and three TRM subsets and annotated them by their most differentially expressed genes. In agreement with previous reports (Qian et al. 2020; Mulder et al. 2021), we differentiated NLRP3 TAMs (*NLRP3*^+^ *IL1B*^+^), SPP1 TAMs (*SPP1*^+^*CHI3L1*^+^*MT1G*^+^), CXCL9 TAMs (*CXCL9*^+^*IL4I1*^+^), and ISG15 TAMs (*ISG15*^+^*CXCL10*^+^*CXCL11*^+^). In addition to the prior published data, we identified three novel TRM subsets: 1) LYVE1^−^FOLR2^+^ TRMs (*FOLR2*^+^*APOE*^+^*TREM2*^+^), 2) LYVE1^+^FOLR2^+^ TRMs (*FOLR2*^+^*LYVE1*^+^*MARCO*^+^*SLC40A1*^+^*SEPP1*^+^) and 3) C3 TRMs (*C3*^+^*CX3CR1*^+^) (Fig 1D).

The existence of two distinct FOLR2 TRMs populations has not been previously reported. Differential gene expression to compare these two subsets showed that FOLR2^+^LYVE1^+^ TRMs were enriched in scavenger receptors (MARCO, CD36, MRC1), metabolic enzymes (BLVRB, PDK4), and immunoglobulins (IGHA1, IGKC, IGLC2). On the other hand, the FOLR2^+^LYVE1^−^ subset was enriched in phagocytosis and antigen presentation gene signatures, further supporting the distinct phenotypes of the two FOLR2-positive populations (Fig 1E).

To explore the distribution of these subsets between CRC and BC, we computed a ratio of their average frequency across samples with more than 35 myeloid cells. CXCL9 TAMs were the most abundant TAM population in both BC and CRC, and NLRP3 TAMs were enriched in CRC, with about 3.5 log2 fold higher frequency than in BC (Fig 1F, S1B–D). Next, we leveraged the fact that the two CRC datasets used (Qian et. al and Lee et. al) contained benign colon samples and compared macrophage cluster distribution across benign and tumor samples. We observed fundamental cluster segregation between benign colon and tumor tissue: NLRP3 TAMs and SPP1 TAMs were almost exclusively confined to colon tumors, whereas LYVE1 TRMs were most enriched in benign colon (Fig S1E–G). Guided by the differential marker gene enrichment between the 11 scRNA Seq myeloid subsets and the differences in their fractional enrichment between normal colon and CRC (Fig 1D, S1E–G) we built a panel of commercially available FFPE-compatible antibodies for six macrophage markers to resolve both TAM and TRM populations (Fig 1G), that consists of IL4I1, NLRP3, SPP1, FOLR2, LYVE1, and MARCO. The following sections describe how we used these markers to discriminate spatial macrophage niches (Fig 2) and to define their cellular composition and function (Fig 3–6).

### FOLR2, IL4I1, NLRP3, and SPP1 mark spatially distinct macrophage niches in the TME.

To study the spatial distribution of macrophage markers in the TME in breast and colon cancer, we used CD68 and CD163 as canonical macrophage markers, IL4I1, NLRP3, and SPP1 to differentiate scRNA TAM subsets, and FOLR2 to highlight TRMs. ScRNA Seq data indicated that NLRP3 is a specific NLRP3 TAM marker, SPP1 is a specific SPP1 TAM marker, but IL4I1 has a broader expression, highlighting SPP1 TAMs, CXCL9 TAMs, and ISG15 TAMs. Nevertheless, the combination of IL4I1, SPP1, and NLRP3 antibodies was sufficient to detect and discriminate NLRP3 TAMs, SPP1 TAMs, and IL4I1 TAM group (encompassing ISG15 and CXCL9 TAMs that we could not resolve) that together labels all scRNA TAMs subsets (Fig 2A). Of note, the Proliferating TAMs are composed of a mixture of cells from different scRNA clusters and form a separate cluster because their gene expression profiles are highly enriched in cell cycle-associated gene expression.

The four panels in Fig 2B–E show staining results of macrophage distribution in a single representative 1.5 mm^2^ tissue region of BC and CRC. Each panel shows 1) an IF image of the discussed markers (*left*), 2) a corresponding dotplot representing the spatial macrophage distribution in the TMA core as revealed by the IF (*top right*), and 3) a corresponding distance quantification from each detected macrophage to the closest tumor cell in that specimen (*bottom right*). We also show distance quantification across a large number of regions and patient samples (Fig 2F, G). We started by analyzing the spatial distribution of CD68 and CD163 (Fig 2B). A commonly held view is that CD163-positive macrophages are of M2-type that help tumor growth and metastasis (Rőszer 2015) and are expected to localize close to tumor cells. Surprisingly, contrary to this view, we found that macrophages with higher CD163 expression (Fig S2A) localized further away from the tumor nests (Fig 2B i, Fig 2F with an average distance of 74.5 μm) compared to macrophages with higher CD68 expression (Fig S2A) that infiltrated and tightly surrounded tumor nests (Fig 2B ii, Fig 2F with an average distance of average 35.9 μm).

Next, we interrogated the spatial distribution of FOLR2, IL4I1, NLRP3, and SPP1 in BC and CRC. We found remarkable and unexpected segregation of these markers where FOLR2 expression was associated with benign tissue localized further away from the tumor (Fig 2C–E). In contrast, macrophages expressing IL4I1 (Fig 2C), NLRP3 (Fig 2D), and SPP1 (Fig 2E) were concentrated immediately adjacent to tumor cells. This was confirmed by a distance comparison that analyzed 36,041 macrophages spanning 60 distinct 1.5 mm^2^ tissue fragments derived from 14 CRC and 13 BC cases. This analysis showed that IL4I1 TAMs were located an average of 38.3 μm away from the closest tumor cell, NLRP3 TAMs 47.4 μm, SPP1 TAMs 36.4 μm, while in contrast, FOLR2 TRMs located 109 μm from the nearest tumor cell (Fig 2G).

Since we found remarkable spatial segregation of IL4I1 TAMs and FOLR2 TRMs in the TME (Fig 2C, G) in primary tumors, we sought to investigate whether this pattern is conserved in metastatic lesions. We compared IF staining of a CRC invasive front and a lymph node CRC metastasis. Similar to the invasive front of the CRC tumor (Fig S2B), in the LN CRC metastasis (Fig S2C), IL4I1 macrophages were present in the desmoplastic stroma surrounding the tumor nests, and FOLR2 macrophages were present further away in the surrounding benign tissue. This suggests that the presence of the tumor shapes macrophage phenotype and distribution in the TME in a similar way independent of the tumor type (BC and CRC share the same TAM populations) and whether the tumor is primary or metastatic. In addition, we report that a thin buffer zone of macrophages co-expressing both FOLR2 and IL4I1 existed in both benign and tumor specimens.

Our results indicate that local tissue cues drive macrophage phenotypes in the spatially segregated tissue areas and suggest that spatially segregated macrophage populations may serve different functions. We show that FOLR2 TRMs are embedded in the normal tissue and are spatially segregated from IL4I1, NLRP3, and SPP1, which are tumor-associated. This is an important finding as revealing markers distinguishing TRMs from disease-associated macrophages is a crucial step that enables the study of individual macrophage subset functions and their relevance to disease progression (Park et al. 2022).

### IL4I1, FOLR2, LYVE1, and MARCO label spatially segregated TRM niches in benign colon and breast.

Next, we sought to learn whether the spatially segregated macrophage distribution we found in the TME was conserved in benign tissue. Previous reports have shown that TRMs govern tissue-specific roles driven by distinct gene expression programs in different normal tissues (Okabe and Medzhitov 2016). However, using our subset-specific markers, we found not one colon-specific TRM population but three distinct layers of TRMs in benign colon mucosa (Fig S2G). We were surprised to find that the IL4I1 macrophages, which we previously discovered to infiltrate tumor nests, were also present in the normal colon mucosa, where they localized at the top of the colon lamina propria (LP) (luminal aspect). The second layer in the middle and bottom of the LP contained FOLR2 TRMs (Fig S2D). The third TRM layer was localized in the colon submucosa and marked by FOLR2, LYVE1, and MARCO (Fig S2E). Since the gastrointestinal submucosa is rich in blood and lymph vessels, the submucosal FOLR2^+^LYVE1^+^MARCO^+^ TRM population likely corresponds to previously reported murine peri-vascular (PV) TRMs (Lim et al. 2018).

In comparison, we found two spatially segregated TRM populations in benign breast stroma. Consistent with a recent report (Nalio Ramos et al. 2022), the TRMs surrounding benign breast lobules and ducts were FOLR2 positive (Fig S2F). We called these cells Lobular TRMs and found they express a dim level of LYVE1 and MARCO (Fig S2F i). Furthermore, we discovered that TRMs localized in the highly vascularized connective tissue that is further removed from the breast lobules co-expressed high levels of FOLR2, LYVE1, and MARCO (Fig S2F ii). We did not detect any IL4I1-positive macrophages in the benign breast stroma (data not shown).

Taken together, these results support the single-cell transcriptomic (Fig S1F) and mIHC (Fig 2D–E,G) findings indicating that NLRP3 and SPP1 macrophages are associated with the TME and FOLR2 and LYVE1 TRMs seed normal tissues. Interestingly, the presence of IL4I1 in both normal colon and CRC suggested that IL4I1 macrophages may seed spatial tissue niches with similar functions rather than being specific to cancerous or normal tissue.

### IL4I1 marks phagocytosing macrophages.

IL4I1 localizes in the lysosomes of antigen-presenting cells (Mason et al. 2004), suggesting a role in phagocytosis. A close inspection of the IF-stained invasive front of colon tumor revealed the presence of pan-cytokeratin (CK)-positive granules within the cytoplasm of IL4I1 TAMs. We hypothesized that the pan-CK granules might be apoptotic bodies derived from tumor cells that are being phagocytosed by the IL4I1 TAMs (Fig 3A). The invasive front of the tumor is an area where intense tissue remodeling takes place. To invade the adjacent normal tissue, tumor cells need to make their way through the wall of tightly joined cells and the extracellular matrix. This process is likely to cause cell death and correlates with a rich presence of IL4I1 TAMs in the CRC invasive front. We also found that the IL4I1 macrophages on the top of the lamina propria in normal colon, but not the FOLR2 TRMs in the middle and bottom of the crypt, contain apoptotic bodies of the intestinal epithelial cells (Fig 3B). Our finding is consistent with work showing that macrophages ingest dying intestinal epithelial cells (IEC) at the top of the intestinal lamina propria (Nagashima et al. 1996) but provides a novel marker for this phenomenon. To further support the hypothesis that IL4I1 marks phagocytosing population of macrophages, we asked whether another specialized body phagocyte type, tingible body macrophages (TBMs), shows IL4I1 positivity. The TBMs localize in germinal centers where they remove apoptotic B cells (Aguzzi, Kranich, and Krautler 2014) and thus are expected to have a high expression of phagocytic markers. TBMs contain apoptotic cellular debris at different degradation stages and are named after apoptotic nuclear debris (‘tingible bodies’) that can be observed in their cytoplasm. We found that the TBM in the LN germinal centers displayed very bright IL4I1 staining (Fig 3Ci) compared to the interfollicular macrophages that were FOLR2 positive (Fig 3Cii). The presence of TBMs is also a hallmark of Burkitt’s lymphoma, a tumor characterized by fast cell turnover (Gotur and Wadhwan 2020). We examined two Burkitt’s lymphoma cases and found that TBMs in this tumor display high IL4I1 expression (Fig 3D).

We used gene set enrichment analysis to further investigate the association between phagocytosis and the IL4I1 TAMs. We found that compared to all other scRNA macrophage subtypes, the CXCL9 TAMs (a subset of *IL4I1*^+^ TAMs) were most enriched in Phagosome, Lysosome, Endocytosis, and Antigen Processing and Presentation gene sets expression (Fig 3E). To evaluate the possible clinical relevance of this finding, we next asked whether IL4I1 TAMs might be targets of phagocytosis-modulating cancer therapies, including anti-CD47 and anti-PD-L1 treatment (Gordon et al. 2017). Notably, using our integrated myeloid object (Fig 1C) we found that *SIRPA* that encodes the ligand for CD47, and *CD274* encoding PD-L1 were both enriched in *IL4I1* expressing scRNA myeloid clusters, including SPP1 TAMs, ISG15 TAMs and CXCL9 TAMs (Fig 3F). This indicates that of all macrophages present in the TME it is the IL4I1 TAMs that likely constitute an indirect target of anti-CD47 and a direct target of anti-PD-L1 immunotherapies. Recent reports demonstrated that PD-L1 expression on TAMs, but not tumor cells, predicts response (Li, van der Merwe, and Sivakumar 2022) and patient survival (Liu et al. 2020) in the context of patients receiving anti-PD-1 axis therapy. Thus, an important question is whether IL4I1 could be used as a predictive marker of response to anti-PD-1 axis blockade. To address this question, we used the scRNA monocyte and macrophage transcriptomes from Bassez et al., dataset (Fig 1C, S1A) that contains samples of advanced breast cancer patients taken before and after pembrolizumab treatment. We found that the frequency of IL4I1 expressing scRNA TAMs, both pre- and post-treatment, increased in patients that responded to the therapy (Fig 3G–H). This important finding suggests IL4I1 as a promising anti-PD-1 axis therapy response marker.

These results 1) demonstrate that IL4I1 is a marker associated with active phagocytosis of individual cells in BC and CRC, 2) suggest that IL4I1 TAMs are targets of anti-CD47 and anti-PD-L1 immunotherapies (Fig 3I) that may affect IL4I1 phagocytosis potential, and 3) indicate IL4I1 as a potential novel predictive marker of PD1-PD-L1 axis blockade.

### CODEX multiplexed imaging reveals spatial cellular interactions in macrophage niches within colon and breast cancer tissues.

Having identified the spatial segregation of the IL4I1, NLRP3, SPP1, FOLR2, and LYVE1 macrophage populations, we sought to elucidate the cellular compositions of the spatially segregated niches where these populations occur. We used CO-Detection by indEXing (CODEX) multiplexed tissue imaging to simultaneously visualize 36 protein markers on a single tissue microarray section of breast and colon benign and tumor tissue (Black et al. 2021; Kennedy-Darling et al. 2021; Goltsev et al. 2018). This panel allowed us to recognize all immune, epithelial and stromal cell types except for neural cells. Our CODEX antibody panel contained four canonical myeloid markers (CD16, CD68, CD163, CD206). To further subtype the macrophage populations, we added SPP1, LYVE1, and FOLR2. Using the CODEX computational pipeline (i.e., imaging processing, single-cell segmentation, and unsupervised clustering) (Hickey, Tan, et al. 2021), we identified two epithelial cell types, seven stromal cell types and fifteen immune cell types (Fig 4A, Fig S3A). Among the immune cell types, we discriminated five macrophage subsets: CD68 TAMs, SPP1 TAMs, CD163 TRMs, FOLR2 TRMs, and LYVE1 TRMs (Fig S3B). We could not add IL4I1, NLRP3, and MARCO antibodies to the CODEX panel for technical reasons. The CODEX-identified CD68 TAMs likely corresponded to IL4I1 TAMs and NLRP3 TAMs populations we identified by IL4I1 and NLRP3 immunostaining in our IF studies. The CODEX-identified CD163 TRMs likely represent LYVE1 TRMs and FOLR2 TRMs for which FOLR2 and/or LYVE1 staining was not detected.

CODEX imaging showed that the distribution of CD68 and CD163 is different between the five macrophage subsets, with CD68 and SPP1 TAMs enriched in CD68 expression while CD163, FOLR2, and LYVE1 TRMs enriched in CD163 expression (Fig S3B). Consistent with the scRNA Seq and 4-color IF results (Fig 1D, Fig S2A–B), CODEX imaging confirmed the existence of 2 FOLR2 positive macrophage populations: FOLR2^+^LYVE1^−^ and FOLR2^+^LYVE1^+^ (Fig S3B). Moreover, we validated that SPP1 TAMs (average distance 28.4 μm) localize more closely to tumor cells than FOLR2 macrophages (average distance 65.8 μm) (Fig S3C). In addition, CODEX data showed that similar to FOLR2 TRMs, the CODEX LYVE1 TRMs are localized further away from the tumor (average distance 106 μm) (Fig S3C).

To uncover the cellular composition of the different macrophage niches, we next performed cellular neighborhood analysis on the CODEX multiplexed imaging data (Schürch et al. 2020; Phillips et al. 2021; Jiang et al. 2022). We clustered cells based on the identity of their ten closest neighboring cells and identified 14 cellular neighborhoods, of which nine were enriched in macrophages (Fig 4A). We grouped the nine macrophage-containing neighborhoods into four neighborhood types, each named after the primary macrophage subtype it contains: 1) CD68 TAM neighborhood, 2) SPP1 TAM neighborhoods, 3) FOLR2 TRM neighborhoods, and 4) LYVE1 TRM neighborhood (Fig 4B). The one CD68 TAM neighborhood was localized inside the tumor nests and co-enriched with the tumor cells (Fig 4B, S4A); we called it the *Intra-tumoral TAM neighborhood*. The three discrete SPP1 TAMs neighborhoods were all enriched with SPP1 TAMs and the tumor cells but differed in cellular composition. The *Peri-tumoral SPP1 TAM neighborhood* contained CD68 macrophages (Fig 4B, S4B), the *Inflamed SPP1 TAM neighborhood* contained neutrophils (Fig 4B, S4C), and the *Hypoxic SPP1 TAM neighborhood* held hypoxic tumor cells marked by CA9 expression (Fig 4B, S4D). The four discrete FOLR2 *neighborhoods* were co-enriched in FOLR2 TRMs and CD163 TRMs but had different cell compositions and tissue locations. *The Plasma Cell (PC) enriched FOLR2 TRM neighborhood* was co-enriched with PCs and located close to the blood vessels and in the normal gastrointestinal (NGI) LP (Fig 4B, S4E). *The Smooth Muscle FOLR2 TRM neighborhood* labeled the bowel muscle wall (Fig 4B, S4F). *The Trapped Fibrous FOLR2 TRM neighborhood* was enriched in FAP fibroblasts and marked fibrous bands entrapped between growing tumor nests (Fig 4B, S4G). The *Lymphoid FOLR2 TRM neighborhood* contained CD4T, CD8T, Tregs, DCs, and FOLR2 TRMs (Fig 4B, S4H). The LYVE1 TRM *neighborhood* was co-enriched with LYVE1 TRMs, FOLR2-TRMs, CD163 TAMs, PDGFRβ fibroblasts, mast cells, and blood and lymph vessels. We called it the *Peri-Vascular LYVE1 TRM neighborhood* (Fig 4B, S4I).

Next, we used two approaches to map each CODEX-macrophage neighborhood tissue distribution relative to the tumor. First, we computed the distance of every macrophage, labeled by the neighborhood it belongs to, to the closest tumor cell (Fig 4C). Second, we calculated the fraction of tumor cells in every macrophage-enriched neighborhood (Fig 4D). We interpret the distance to the tumor and the fractional enrichment in tumor cells as an indicator of how closely the given neighborhood is associated with the tumor. These analyses revealed a remarkable spatial macrophage neighborhood segregation and a 3-tier distribution of closeness to the tumor. Specifically, we show that TAMs in *the Hypoxic SPP1 neighborhood* and *the Intra-tumoral neighborhood* were located the closest to the tumor cells with an average distance of 9.37 and 10.6 μm to the nearest tumor cell (Fig 4C) and that those two neighborhoods had the highest fraction of tumor cells (Fig 4D). In contrast, TRMs in *the Lymphoid FOLR2*, *the PCs enriched FOLR2, the Peri-Vascular LYVE1* and *the Smooth Muscle FOLR2 neighborhoods* lay the farthest from the tumor with an average distance of 55.2, 57.5, 74.8, and 76.0 μm from the closest tumor cell (Fig 4C). In agreement, they also contained the smallest percentage of tumor cells (Fig 4D). Macrophages in *The Peri-tumoral SPP1, the Inflamed SPP1,* and *the Trapped Fibrous FOLR2 neighborhoods* localized at an intermediate distance between the two extremes.

To better visualize the spatial distribution of the macrophage neighborhoods in benign and tumor tissues, we plotted the neighborhood frequency by anatomic location. We show that *the Peri-Vascular LYVE1 TRMs neighborhood* was most enriched in benign breast, while *the PCs enriched FOLR2 TRMs neighborhood* was most enriched in NGI mucosa. *The Smooth Muscle FOLR2 TRMs neighborhood* labels bowel wall and was thus specific to gut samples, and it could be detected in benign, in the invasive front and center of the tumor samples. This is consistent with the fact that CRC invades the bowel wall. In turn, the *Intra-tumoral TAM neighborhood, the Inflamed SPP1 TAM neighborhood, the Peri-tumoral SPP1 TAM neighborhood, the Hypoxic SPP1 TAM neighborhood, and the Trapped Fibrous FOLR2 neighborhood* were enriched in ductal carcinoma in situ (DCIS), invasive ductal carcinoma (IDC), in the IF of CRC and the CRC center of the tumor (CT), further supporting that they are tumor-associated (Fig 4E).

Taken together, the CODEX data (Figs 4, S3, S4) allowed us to identify spatial associations between macrophage subtypes and other cell types in benign and tumor tissues. We showed that SPP1 TAMs were co-enriched with CD68 TAMs close to the tumor cells, localized in hypoxic tumor areas, and associated with neutrophilic infiltration. In contrast, CD163 TRMs, FOLR2 TRMs, and LYVE1 TRMs were co-enriched in adjacent benign tissue located further away from the tumor. We showed that FOLR2 TAMs constituted a tissue-resident macrophage population in the bowel muscle wall and were associated with PCs in the intestinal lamina propria and connective breast tissue. We found that FOLR2 TRMs from the breast connective tissue or muscle bowel wall can be trapped within growing tumor nests and thus become a part of the TME (Fig 4F).

### FOLR2 TRMs spatially colocalize with plasma cells and may maintain long-lived plasma cell tissue niche.

To further explore the CODEX-identified FOLR2 TRM association with PCs, we used IHC and multicolor IF. Single color IHC showed that in the tumor-adjacent stroma FOLR2 TRMs were in direct contact with PCs, which can be histologically identified by their nuclear chromatin condensation pattern and asymmetric cytoplasmic ‘hof’ where antibodies are produced and stored (arrowheads, Fig 5A). To unequivocally demonstrate that the cells spatially co-enriched with FOLR2 TRMs were PCs, we used 4-plex IF staining and showed that cells localized directly next to FOLR2 TRMs were marked by overlapping expression of CD38 and a prototypical PC marker - CD138 (Fig 5B). Multicolor IF additionally revealed that FOLR2 TRMs and CD38^+^ PC occupied the same space in the middle and bottom layers of the colon lamina propria (Fig S5A left panel), corroborating the CODEX results. Furthermore, we found Lobular FOLR2 TRMs were immediately adjacent to PCs around benign breast glands (Fig S5A right panel). Previous studies showed that CD163^+^ macrophages surround PCs in the extrafollicular foci in the tonsil (Xu et al. 2012). Here we show that it was the FOLR2 TRM subtype that localized directly next to PCs in the LN interfollicular zone (Fig S5B).

To demonstrate that the association between PCs and FOLR2 TRMs was specific, we computed the distance from every IL4I1 TAM and FOLR2 TRM to their closest PC across seven different tissue regions. As anticipated, PCs were localized closer to FOLR2 TRMs than the IL4I1 TAMs (Fig 5C–D).

To gain insight into the possible molecular mechanism governing the contact between the FOLR2 TRMs and PCs, we next performed scRNA Seq-based ligand-receptor interaction analysis using published data from two studies. First, we used PCs and FOLR2 TRMs transcriptomes from the scRNA Seq study on BC patients (Bassez et al. 2021). The highest probability interactions were found between APRIL (TNFSF13) and BAFF (TNFSF13B) on the FOLR2 TRMs and BCMA (TNFRSF17) on the PCs (Fig 5E). APRIL and BAFF are known to drive PC infiltration and their long-term survival in the tissue (Kawakami et al. 2019; Benson et al. 2008). Similarly, using the IgA^+^PC, IgG^+^PC, and FOLR2 TRMs scRNA Seq transcriptomes from benign colon and CRC (H.-O. Lee et al. 2020), we also identified BAFF (TNFSF13B) and BCMA (TNFRSF17) interaction as the highest probability interaction between IgA^+^PC and FOLR2 TRMs (Fig S5C). Our results provide a marker for the type of macrophage described in previous literature that suggests antigen-presenting cells maintain the PC niche in human tonsils (Xu et al. 2012), murine bone marrow (Rozanski et al. 2011), and human lamina propria (Hickey, Becker, et al. 2021). Taken together, these observations suggest that FOLR2 TRMs play a key role in recruiting and maintaining PCs in inflamed benign tissue adjacent to tumors and the lamina propria of benign colon (Fig 5F).

### SPP1 TAMs seed hypoxic and necrotic tumor areas and NLRP3 TAMs activate NLRP3 inflammasome in the TME

CODEX neighborhood analysis revealed spatial co-enrichment of SPP1 TAMs with neutrophils in *the Inflamed SPP1 TAM niche*. Notably, we also found NLRP3 TAMs to be enriched in neutrophil-infiltrated tumor areas (Fig 6A). However, unlike NLRP3 TAMs, which were spatially co-enriched with live neutrophils in viable areas, SPP1 TAMs were associated with areas containing necrotic tissue (Fig 6B). This observation prompted us to compare NLRP3 and SPP1 TAMs’ transcriptomes. Differential gene expression showed that NLRP3 TAMs expressed high levels of neutrophil chemoattractant cytokines (*CXCL1*, *CXCL2*, *CXCL8*). In contrast, the most upregulated genes in SPP1 TAMs were associated with phagocytosis and lipid metabolism, including apolipoproteins (*APOC1, APOE*), lipid scavenger receptors (*TREM2, MARCO*), lipid transporter *FABP5,* cathepsins (*CTSB, CTSD, CTSZ*), and matrix metalloproteinase (*MMP9, MMP12*) (Fig 6C–D). Interestingly, SPP1 itself has been implicated in phagocytosis (Shin et al. 2011; Schack et al. 2009) and lipid metabolism (Remmerie et al. 2020). To further interrogate the association of SPP1 TAMs with necrosis, we used a publicly available 10x Visium FFPE Human Breast Cancer sample to show that necrotic tumor areas in this specimen were enriched in SPP1 rather than NLRP3 gene expression (Fig 6E). These results suggest that NLRP3 TAMs likely contribute to neutrophil recruitment in the TME and that the SPP1 TAMs may play a role in the phagocytosis of necrotic tumor. It is important to note that although both IL4I1 TAMs and SPP1 TAMs are associated with phagocytosis and the SPP1 TAMs are a subset of IL4I1 TAMs, the IL4I1 macrophages seed viable tissue areas that are enriched in cells undergoing individual cell death, whereas the SPP1 TAMs are enriched in areas with large regions of hypoxic and necrotic tissue that is characterized by the presence of deceased neutrophils.

NLRP3 is a pathogen and danger-associated molecular pattern receptor known to form an intracellular complex called the inflammasome, leading to proteolytic pro-IL1β activation and release. IL1β is known to play a role in neutrophil recruitment in infection (Miller et al. 2007) and cancer (Chen et al. 2012). Inflammasome activation results in the assembly of proteins forming an inflammasome into a micrometer-sized protein complex called a speck (Lamkanfi and Dixit 2014). Speck formation can be used as a simple readout for inflammasome activation (Stutz et al. 2013). Interestingly, we observed that in breast and colon cancer, the NLRP3 expression could be either seen as a diffuse expression within the macrophage cytoplasm (Fig 6F i) or aggregated in a single speck (Fig 6F ii). We found that speck-like NLRP3 aggregation, which we interpret as activated inflammasome complexes, was linked to neutrophil infiltration (Fig 6F ii). To confirm, we stratified BC and CRC NLRP3 TAM-positive regions by whether they were enriched in NLRP3 TAMs with diffuse staining or NLRP3 TAMs with NLRP3 specks, and quantified the number of neutrophils. The presence of NLRP3 specks in the cytoplasm of macrophages correlated significantly with neutrophil tissue infiltration (Fig 6G). Thus, we hypothesize that assembly of the inflammasome in NLRP3 TAMs likely induces IL1β activation and secretion, which drives neutrophil infiltration (Fig 6H).

To extend our findings beyond cancer, we investigated whether we could detect NLRP3 inflammasome activation in Crohn’s Disease (CD), a type of inflammatory bowel disease associated with neutrophil infiltration. Indeed, the examination of three cases of advanced CD showed that regions with high macrophage infiltration 1) contained macrophages with NLRP3 specks and 2) were highly infiltrated by neutrophils (Fig S6A–B). The most convincing human studies implicating inflammasome involvement in human cancer are based on SNP associations and a report that IL1β blockade in atherosclerosis correlated with reduced incidence of lung cancer (Ridker et al. 2017; Sharma and Kanneganti 2021). We are the first to provide histologic evidence demonstrating inflammasome formation in human BC, CRC, and CD in human FFPE tissue sections and to demonstrate an association of the NLRP3 inflammasome formation with neutrophil infiltration.

Previous reports showed that macrophage subtype signatures, including that of SPP1 TAM (Zhang et al. 2020; H.-O. Lee et al. 2020) and FOLR2 TRM (Nalio Ramos et al. 2022), are predictive of clinical outcome in cancer. However, the association of macrophage niches (understood as a collection of spatially interacting cells) with clinical outcomes remains largely unexplored. We, therefore, determined the prognostic association of gene signatures of *Neutrophil and NLRP3 TAM* and *Neutrophil* and *SPP1 TAM* niches in clinically-annotated datasets, including the PRECOG data (Gentles et al. 2015). In this analysis, the enrichment of the *Neutrophil and SPP1 TAM Niche* gene signature is a surrogate for the hypoxic and necrotic SPP1 TAM Niche, and the enrichment of the *Neutrophil and NLRP3 TAM Niche* gene signature is a surrogate for NLRP3 inflammasome activation that we found to correlate with neutrophil infiltration in the TME. In addition, we interrogated a *FOLR2*/*SEPP1*/*SLC40A1* gene signature, previously associated with favorable clinical outcomes in BC, as a reference. In line with previous reports (Ramos et al. 2022) and corroborating our approach, we found that the *FOLR2*/*SEPP1*/*SLC40A1* signature predicted favorable outcomes in BC but not CRC (Fig 6I, S6C–D). This analysis also showed that *SPP1 TAM* gene signature expression and enrichment of the *Neutrophil and SPP1 TAM Niche* gene signature were strong predictors of poor outcome in BC and CRC. This is consistent with the spatial associations of SPP1 TAMs and tumor necrosis and hypoxia (Fig 4B, S4D, 6B, 6E), and the fact that both tumor necrosis and hypoxia are hallmarks of tumor aggressiveness (Lam et al. 2005; Swinson et al. 2002; Fisher et al. 1993). Interestingly, we found that while the *NLRP3 TAM* gene signature expression alone did not correlate with BC or CRC patient outcomes, the enrichment of the *Neutrophil and NLRP3 TAM Niche* gene signature was strongly associated with adverse BC outcomes. This reflects our IF findings showing that NLRP3 protein TAM expression alone is not spatially associated with neutrophils, while NLRP3 inflammasome assembly in a speck correlates with neutrophil tissue infiltration (Fig 6F–H). In this instance, the *NLRP3 TAM* gene signature reflects the diffuse NLRP3 protein expression in the cell (Fig 6Fi), and the *Neutrophil and NLRP3 TAM Niche* gene signature correlates with the NLRP3 inflammasome activation that shapes the tumor inflammation by neutrophil tissue recruitment (Fig 6Fii). Thus, this finding indicates that NLRP3 inflammasome activation is associated with worst BC outcomes.

Taken together, these results suggest that NLRP3 TAMs may be involved in the onset of inflammation by activating the NLRP3 inflammasome and may be driving neutrophil infiltration in the TME and Crohn’s Disease. In addition, we demonstrate that the abundance of *Neutrophil inflamed NLRP3 TAM Niche* is associated with poor BC patient outcomes, suggesting that NLRP3 targeting in cancer might be a novel and promising treatment avenue.

## Discussion

This work reveals a rich landscape of spatially segregated functional macrophage niches across malignant human breast and colon tissue with correlates in normal tissue in these organs. We demonstrate that macrophage niches are not specific to an anatomical location or disease but rather conserved between tissue compartments with similar local cues. For example, IL4I1 macrophages are embedded in areas enriched in individual cell death in the desmoplastic stroma at the invasive front of the tumor, the colonic upper lamina propria, and LN germinal centers. Thus, our findings indicate that macrophage niches are fundamental functional building blocks of tissue. In addition, we uncover some of the incoming and outgoing signals governing the macrophage niche. For example, we are the first to histologically identify NLRP3 inflammasome activation in human cancer and to show that it is associated with neutrophil recruitment.

It has been recognized that TRMs across different organs exhibit specialized functions reflecting local tissue physiology (Okabe and Medzhitov 2016). However, we are the first to uncover the existence of distinct functional spatial niches harboring discrete macrophage populations and cellular compositions within a single organ system. In particular, we reveal the existence of four separate macrophage niches in the bowel wall, including a phagocytic IL4I1 TAM niche, a novel FOLR2 TRMs plasma cell niche, a perivascular LYVE1^+^FOLR2^+^ TRMs niche in the bowel submucosa, and a smooth muscle FOLR2 TRMs niche in the muscularis propria.

Notably, our results reveal that IL4I1, SPP1, and NLRP3 TAM niches are closely associated with the tumor nests and implicated in the cancer response, including individual tumor cell death, hypoxia and diffuse tissue necrosis, and acute inflammation, respectively. In addition, IL4I1 TAMs might be implicated in response to anti-CD47 and anti-PD-L1 therapy as they express the CD47 ligand- *SIRPA* and *CD274* encoding PD-L1, and correlate with anti-PD1 treatment response. Moreover, we show that NLRP3 inflammasome activation correlates with acute inflammation in BC, CRC, and CD and is associated with adverse patient outcomes in BC. This finding nominates the NLRP3 inflammasome as a novel therapy target where its specific small molecule inhibitor - MCC950 (Coll et al. 2015) could function as a novel therapeutic agent in solid tumors and CD.

Collectively, our findings elucidate a landscape of discrete human macrophage niches, uncover unexpected cell interactions and mechanisms governing the macrophage niche biology, explore the prognostic significance, and suggest novel therapy targets. Importantly, since the tools we present are FFPE-compatible, they enable the use of archival clinical material and provide a framework for the study of human macrophage function in health and disease.

### Limitations of the study

Ideally, macrophage tissue distribution and function should be profiled by simultaneous visualization of all macrophage populations. However, we could not include IL4I1 and NLRP3 antibodies for CODEX imaging due to the incompatibility of working FFPE clones with DNA tags for adequate staining. In effect, we detected a large population of CODEX CD68 TAMs that localize close to the tumor and likely correspond to the IL4I1 and NLRP3 TAMs we characterize using the IF. Additionally, the evidence we present to propose the function of the discrete macrophage population is based on gene expression and imaging observations. Thus our findings warrant and inform functional studies to validate our observations.

## Methods

### RESOURCE AVAILABILITY

#### Lead Contact

Further information and requests for resources should be directed to and will be fulfilled by the Lead Contacts Magdalena Matusiak (mmatusia@stanford.edu) and Matt van de Rijn (mrijn@stanford.edu).

#### Materials Availability

This study did not generate new unique reagents.

#### Data Availability

Publicly available scRNA Seq datasets analyzed in this study are available under following links: Qian et al. (Qian et al. 2020) and available under https://lambrechtslab.sites.vib.be/en/pan-cancer-blueprint-tumour-microenvironment-0, CRC data from Lee et al. (H.-O. Lee et al. 2020) available in the NCBI Gene Expression Omnibus (GEO) database under the accession codes GSE132465, GSE132257 and GSE144735, and data from Bassez et al. (Bassez et al. 2021) available at https://lambrechtslab.sites.vib.be/en/single-cell. The spatial transcriptomic array with Human Breast Cancer: Ductal Carcinoma In Situ, Invasive Carcinoma (FFPE) sample data is available from 10x website https://www.10xgenomics.com/resources/datasets/human-breast-cancer-ductal-carcinoma-in-situ-invasive-carcinoma-ffpe-1-standard-1-3-0

### EXPERIMENTAL MODEL AND SUBJECT DETAILS

#### Human Patient Samples

All clinical specimens in this study were collected with informed consent for research use and were approved by the Stanford University Institutional Review Boards in accordance with the Declaration of Helsinki.

##### Breast and colon cohorts FFPE samples

This study used FFPE samples from 36 invasive breast cancer (IBC) and 32 colon carcinoma (CRC) cases.

##### Crohn’s Disease FFPE samples

We performed the analysis in Fig 6A–B, using three advanced Crohn’s Disease patient FFPE samples.

##### IF and CODEX Tissue microarrays

The tissue microarrays used in ths study were constructed from 36 1.5 mm^2^ regions from 19 CRC cases, and 29 1.5 mm^2^ regions from 18 IBC cases. Regions were selected based on differential spatial staining observed on full section staining with IL4I1, SPP1 and FOLR2 antibodies.

### METHOD DETAILS

#### External datasets

##### Single-cell RNA-seq tumor atlases

We obtained preprocessed scRNA-seq count data from four datasets covering breast carcinoma (BC), and colon carcinoma (CRC). Specifically we used CRC and BC datasets published by Qian et al. (Qian et al. 2020), CRC data from Lee et al. (H.-O. Lee et al. 2020), and BC data from Bassez et al. (Bassez et al. 2021). For each dataset, we extracted monocytes, macrophages, and dendritic cells by clustering SCTransformed count data using Seurat and subsetting clusters expressing AIF1, CST3, CD68, CD163, ITGAX, and HLA-DRA. Next, we integrated the myeloid clusters from the 4 datasets using the reciprocal PCA workflow with Seurat. We used log normalization. To clean the data we excluded dying cells, stressed cells, and cell duplets. We identified dying cells’ clusters by inspecting the distribution of log2(nCount_RNA+1) per cell. Stressed cells were identified based on high expression levels of HSP genes. Cell duplets were identified based on the coexpression of non-myeloid cell markers as follows: myeloid-epithelial cell (TFF3, keratins), myeloid-Tcells (*CD3D*), myeloid-stromal cells (*SPARCL1, SPARC, COL1A1*), and myeloid-plasma (immunoglobulin genes). Since we intended to focus exclusively on monocytes and macrophages, we excluded neutrophils and dendritic cell clusters identified based on the following gene enrichment: neutrophils (SOD2, GOS2, and low detected number of counts per cell), cDC1s (*CLEC9A*), cDC2s (*FCER1A, CD1C, CD1E, and CLEC10A*), migratoryDC (*BIRC3, CCR7, LAMP3*), follicular DC (*FDCSP* and immunoglobulin genes), plasmacytoid DC (*JCHAIN, LILRA4, IRF7*), CD207^+^ DC (*CD1A, CD207, FCAR1A*). Next, we re-clustered the integrated and cleaned Seurat object containing only monocytes and macrophages with resolution = 0.6 in the *FindClusters()* function. We obtained 15 clusters and annotated them based on the most differentially expressed genes in each cluster. Monocytes have been identified by *FN1*, *FCGR3A*, and *VCAN*. Macrophages were identified based on *C1QA*, *APOE*, and *TREM2* expression. We merged clusters 0 and 12 into ISG15 TAMs, clusters 1, 6, and 14 into CXCL9 TAMs, and clusters 11 and 13 into LYVE1^+^FOLR2^+^ TRMs. The resulted myeloid object is presented in Fig 1C.

##### Spatial transcriptomics

We obtained pre-processed spatial transcriptomic data from Human Breast Cancer: Ductal Carcinoma In Situ, Invasive Carcinoma (FFPE) sample data from 10x website https://www.10xgenomics.com/resources/datasets/human-breast-cancer-ductal-carcinoma-in-situ-invasive-carcinoma-ffpe-1-standard-1-3-0 (Fig 6E).

##### Clinically-annotated tumor transcriptomes

We analyzed 4.231 pre-normalized carcinoma transcriptomes of BC and CRC from the Prediction of Cancer Outcomes using Genomic Profiles (PRECOG) database (Gentles et al. 2015), along with additional datasets listed in Table S4, all of which were processed according to the PRECOG workflow (Gentles et al. 2015). Only datasets with at least 25 samples and available overall survival data were included (Table S4). Specifically, we analyzed 3.905 BC patient samples from 16 datasets and 326 CRC patient samples from 4 datasets.

#### Enrichment of monocyte and macrophage scRNA Seq populations

For the analysis in Figs 1F, S1C–D,F–G, we selected samples with more than 35 monocyte and macrophage cells and computed the frequency of the different scRNA subsets in each sample. Figs S1C–D,F, we present these frequencies stratified by tumor type and anatomical location. In addition, for Fig 1F and S1F, we computed a mean frequency for every scRNA subset and calculated a ratio of its frequency between BC and CRC (Fig 1F) and normal colon and CRC (Fig S1F).

#### Average cluster gene expression

The average gene expression dotplots per scRNA monocyte and macrophage clusters in Fig 1D, 2A, 3F, 6D were plotted using the aggregated myeloid object from Fig 1C.

#### Spatial transcriptomics dataset processing and visualization

For the analysis in Fig 6E, we used STutility r package to normalize, annotate and visualize the pre-processed spatial transcriptomic data. Specifically, we used the SCTransform function for normalization and the ManualAnnotation function to annotate data based on the H&E image.

#### Immunohistochemistry

For the analysis in Fig 5A and 6A–B, 4 μm tissue sections were deparaffinized and rehydrated. Subsequently, antigen retrieval was performed in EDTA pH 9 buffer for 5 min at 95 °C in a pressure cooker. Slides were next stained with FOLR2, SPP1 or NLRP3 antibodies listed in Table S1, and imaged with a Keyence BZ-X800 microscope at 20´ magnification.

#### Immunofluorescence (IF)

For the analyses shown in Fig 1G, 2B–E, S2B–F, 3A–D, 5B, S5A–B, 6F, S6A 4μm full tissue sections were deparaffinized and rehydrated. Antigen retrieval was performed using EDTA pH 9 buffer at 95 °C for 10 min. Sections were blocked for 20 min with horse serum and stained for 1h with primary antibodies. Sections were subsequently stained with secondary antibodies for 1 h. A list of primary and secondary antibodies used in this work can be found in Table S1. Sections were then mounted in ProLong Gold Antifade reagent with DAPI and cover-slipped. Stained sections were imaged with a Keyence BZ-X800 microscope at 20´ or 40’ magnification. Of note, LYVE1 is expressed on both TRMs and lymphatic endothelial cells. Yet, lymphatic endothelial cells can be readily differentiated from TRMs as they are organized in tubes, display much higher LYVE1 expression than TRMs, and do not express FOLR2 and MARCO (Fig S2D).

#### IF images dearraing

IF images were acquired with a Keyence BZ-X800 microscope at 20´ magnification. Next, the TMA core coordinates were extracted using the dearray functionality in QuPath (Bankhead et al. 2017). Subsequently, the TIFF TMA images were dearrayed using QuPath extracted core coordinates with vips crop function in Linux command line.

#### IF images cell segmentation and immunofluorescence signal quantification

Cell nuclei on the dearrayed TMA cores were segmented using Mesmer (Greenwald et al. 2022). Subsequently, IF signal was quantified for each detected nuclei by computing staining intensity within 3-pixel distance from the nuclear border. We consider a nucleus and its accompanying IF signal within 3-pixel distance from the nuclear border as a cell. In effect, each cell is described by its x and y pixel coordinate and IF staining intensity.

#### Clustering and annotation of IF data

Each individual IF staining was clustered separately. First, IF staining intensity was z normalized using zscore function from scipy.stats python module. Next, cells were clustered using Leiden clustering implementation in scanpy python package. All clusters were individually visually inspected on the dearrayed TIFF images by indicating location of cells attributed to a given cluster. Cell clusters were annotated based on morphology, location, and staining intensity.

For Fig 5C–D, we clustered and annotated cells form 7 1.5 mm^2^ tissue regions including 6 BC and 1 CRC cases. We used FOLR2, IL4I1, and CD138 staining intensity to discriminate FOLR2 TRMs, IL4I1 TAMs and PCs, respectively.

#### Distance quantification of IF and CODEX data

For every TMA core, the distance between every cell and every other cell present in the core was computed using cdist function from scipy.spatial.distance python module. Next, for every macrophage, the shortest distance to a Tumor Cell was selected from the matrix of all cell distances. This shortest distance is reported as the distance to the closest Tumor Cell. For CODEX data, normal breast and gastrointestinal tract samples were excluded.

##### Significance assessment within one tissue region

Wilcox test was used to assess the significance in Figures 2B–E.

##### Significance assessment across multiple tissue regions

Linear mixed-effect models were used to assess significance in Figures 2F–G, 4C, S3C, 5D. We used the lmer function from package lme4 (v1.1.21), and took the tissue region intercept as a random effect. The pairwise p-values were derived from t-ratio statistics in the contrast analysis using the lmerTest (v3.1.2) and corrected for multiple hypothesis testing using the Holm Bonferroni method implemented in the modelbased (v0.1.2) package (github.com/easystats/modelbased).

#### CODEX macrophage distance quantification by niche

For distance quantification in Fig 4C, macrophages were stratified by the macrophage niche they belong to.

#### CODEX antibody panel

The antibody panel in this study was constructed by selecting antibodies targeting epithelial and stromal tumor compartments, with a focus on the myeloid compartment. Detailed information on the included antibodies can be found in Table S2. Each antibody was first conjugated to a unique oligonucleotide tag. Next, antibody-oligonucleotide conjugates were tested in low-plex fluorescence assay to determine whether their staining patterns match patterns established in IHC and IF experiments and to establish the best staining concentration and exposure time. Subsequently, all antibody conjugates were tested together in a single test CODEX imaging multicycle to evaluate optimal concentration, exposure time, and imaging cycle.

#### CODEX imaging

CODEX imaging was performed as previously described (Black et al. 2021). BC and CRC tissue microarrays were simultaneously stained with a previously validated cocktail of antibody-oligonucleotide conjugates and sequentially subjected to CODEX multiplexed imaging using the optimized conditions established during the test run. Metadata with detailed information on each CODEX run can be found in Table S3.

#### CODEX data processing

CODEX imaging data was processed using a software tool called RAPID (Lu G, et al. Manuscript under review, 2022), which included 3D GPU-based deconvolution, spatial drift correction, image stitching, and background subtraction (available at https://github.com/nolanlab/RAPID). Next, cell nuclei segmentation on the processed images was performed using a neural network-based segmentation algorithm called CellVisionSegmenter. CellVisionSegmenter has been shown to work well with segmenting both dense and diffuse cellular tissues with CODEX data (M. Y. Lee et al. 2022). CellVisionSegmenter is an open-source, pre-trained nucleus segmentation and signal quantification software based on the Mask region-convolutional neural network (R-CNN) architecture. The only parameter that was altered was the growth pixels of the nuclear mask, which we found experimentally to work best at a value of 3.

#### CODEX data clustering, visualization, and cell type assignment

Cell clustering and annotation were performed according to a previously published protocol (Hickey, Tan, et al. 2021). First, nucleated cells were selected by subsetting cells with positive Hoechst signal imaged in 2 separate CODEX cycles. Next, marker signal intensity was z-normalized, and data was overclustered using Leiden clustering in scanpy Python package. Each cluster was visually examined by mapping a location of cells attributed to a given cluster to processed CODEX images and inspecting its marker staining. ImageJ was used to view processed CODEX images. Cell clusters were annotated based on cell morphology, tissue location, and marker staining intensity.

#### CODEX niche analysis

Niche analysis was performed as described earlier by Schurch et al. (Schürch et al. 2020) with k = 10 nearest neighbors and 30 clusters. The cell clusters were annotated and grouped into 13 Niches based on location in the tissue and cell type enrichment score.

#### Ligand-Receptor interaction analysis

Ligan-Receptor analysis was performed using CellChat R package workflow with default settings and using netVisual_bubble function to extract all identified significant liganr-receptor interactions between FOLR2 TRMs and Plasma Cells (PCs). For the analysis in Fig S5C, IgA^+^ and IgG^+^ PCs annotation was extracted from Lee et al. (H.-O. Lee et al. 2020). For Fig 5E, PCs were identified using FindClusters Seurat function with res = 0.4, and selecting cluster #19 with high CD38 and JCHAIN expression. Fig 5E shows all detected significant interactions between FOLR2 TRMs and PCs. Fig S5C shows 10 top significant interactions detected between FOLR2 TRMs and IgA^+^ and IgG^+^ PCs.

#### Gene Set Enrichment Analysis

KEGG pathway gene set enrichment analysis from Fig 3E was performed using clusterProfiler R package. The KEGG enrichment was performed on the list of differentially enriched genes between the 11 transcriptional MAC scRNA Seq populations. Next, enrichment results of Antigen processing and presentation, Phagosome, Lysosome, and Endocytosis gene sets were plotted to compare enrichment of phagocytosis-related pathways between the scRNA MAC populations.

#### Pembrolizumab response analysis

For the analysis in Fig 3G–H, was performed on scRNA myeloid transcriptomes form Bassez et al., that we subseted from the aggregated myeloid object form Fig 1C. The patient samples were stratified by the authors of the oryginal publication based on whether the T cell repertoire, as assessed by TCR sequencing, expanded (E) or not (NE) after the pembrolizumab administration. We labeled patients with expanded T cell repertoire as responders (R) and patients with non-expanded T cell repertoire as non-responders (NR).

For the analysis in Fig 4H, we used scRNA monocyte and macrophage transcriptomes of responders and non-responders pre pembrolizumab treatment. We first computed scRNA cluster frequencies in patients with more than 35 monocyte and macrophage cells. Next we compared the mean scRNA cluster frequencies with Chi-squared test using chisq.test function from stats R package and used chisq.posthoc.test function from chisq.posthoc.test R package to asses significance. p values were adjusting using Bonferroni correction.

#### Neutrophil infiltration quantification in BC, CRC, and Crohn’s Disease

For the analysis in Fig 6G, we counted the number of neutrophils present in 1.5 mm^2^ tissue microarray (TMA) cores. The IF-stained TMA cores were evaluated by a pathologist and stratified into cores containing CD68 positive macrophages with diffuse NLRP3 staining or cores that contained CD68 positive macrophages with NLRP3 aggregated in a speck. Cores that contained both diffused and aggregated NLRP3 were classified as cores with NLRP3 speck, as we assumed that the NLRP3 aggregation contains active inflammasome complex that projects the inflammatory signaling. For the analysis in Fig S6B, we counted the number of neutrophils in 1mm^2^ tissue regions selected from whole slide sections. We selected areas that contained CD68 positive macrophages containing NLRP3 aggregated in a speck. Since we didn’t detect any macrophages with NLRP3 diffused staining in the Crohn’s disease tissue sections, we compared the neutrophil numbers in Crohn’s disease patients to benign colon submucosa. CD68 and NLRP3 signals were used to identify NLRP3 TAMs, and Calprotectin was used to identify neutrophils.

#### Survival analyses

For the analyses in Fig 6I and S6C–D, we applied univariable Cox proportional hazards regression to link the relative enrichment of each gene signature (Table S5) to overall survival (survival R package v2.42.3 (Therneau and Grambsch, 2000)) and integrated the resulting z-scores across datasets of the same tumor type as described in (Luca et al. 2021). All survival z-scores were converted to two-sided −log10 p values for clarity.

### QUANTIFICATION AND STATISTICAL ANALYSIS

Wilcoxon test was applied for group comparisons. Linear mixed effect models were applied when groups contained multiple observations from the same tissue region (for instance, when comparing the distance of macrophages to tumor cells across multiple tissue regions). Results with *P* < 0.05 were considered significant. Error bars on the bar plots represent standard deviation (SD). Data analyses were performed with R and python. The investigators were not blinded to allocation during experiments and outcome assessment. No sample-size estimates were performed to ensure adequate power to detect a pre-specified effect size.

## Supplementary Material

Supplement 1

## Figures and Tables

**Fig 7.**
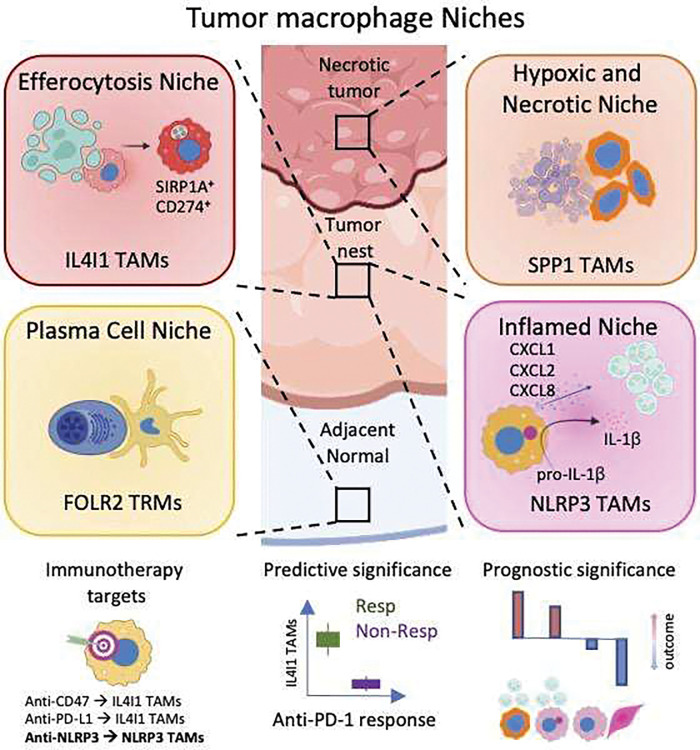
Macrophages in the TME IL4I1 TAMs, SPP1 TAMs and NLRP3 TAMs are infiltrating tumor microenvironment and FOLR2 TRMs are localized in the tumor adjacent benign tissue. The IL4I1 TAMs are enriched in tissue niches with high cell turnover where they perform efferocytosis. The SPP1 TAMs seed necrotic and hypoxic tumor areas where they clean dead tissue fragments. The NLRP3 TAMs shape the inflamed tumor niche by NLRP3 inflammasome activation and resulting neutrophil recruitment. In addition, IL4I1 TAMs are likely targets of anti-CD47 (indirect target) and anti-PD-L1 (direct target) immunotherapies as they express *SIRPA* (encoding CD47 ligand) and *CD274* (encoding PD-L1). IL4I1 TAMs may serve as predictive marker as they are associated with response to anti-PD1 therapy. In turn, the NLRP3 inflammasome activation and resulting neutrophil tissue infiltration corelates with adverse outcome in breast cancer rationalizing NLRP3 inflammasome activation targeting in breast cancer.
