# Supplementary material for: A spatial map of human macrophage niches reveals context-dependent macrophage functions in colon and breast cancer
Source: Res Sq. 2023 Jan 10:rs.3.rs-2393443. Preprint. [Version 1] doi: 10.21203/rs.3.rs-2393443/v1 (PMC9882614; doi:10.21203/rs.3.rs-2393443/v1)
Supplement: Supplement 1 [file NIHPPrs2393443v1-supplement-1.pdf]

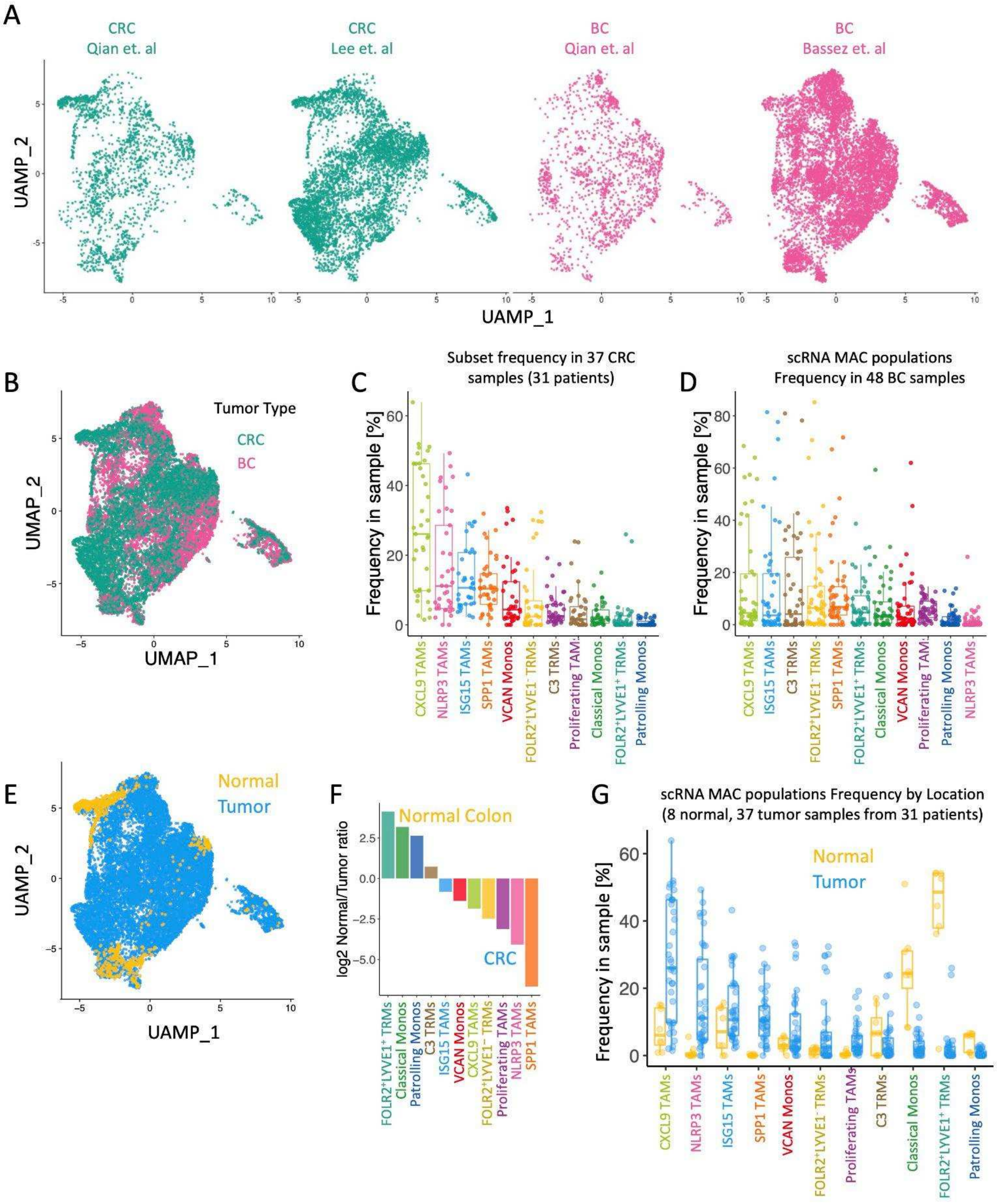

Fig S1. ScRNA Seq reveals differences in spatial enrichment of myeloid markers, related to Fig 1
(**A**) UMAP projection of monocytes and macrophages scRNA transcriptomes grouped by and colored by dataset showing the contribution of each dataset. (**B**) UMAP projection of monocytes and macrophages scRNA transcriptomes colored by tumor type. (**C)** Boxplots show the frequency of scRNA macrophage populations across 37 samples in 31 CRC patients ordered by their average expression. (**D**) Same as (**C**) but in 48 BC patients. (**E**) UMAP projection of monocyte and macrophage scRNA transcriptomes from 4 studies colored by normal vs. tumor specimens. (**F**) Barplot of the ratio of log2 average fractional scRNA myeloid population enrichment between Normal colon samples and CRC samples in 2 CRC scRNA Seq datasets (H.-O. Lee et al. 2020; Qian et al. 2020). (**G**) Same as (**C**) but in 8 normal colon samples and 37 CRC samples in 31 CRC patients and ordered by average frequency of cell populations in Tumor samples.

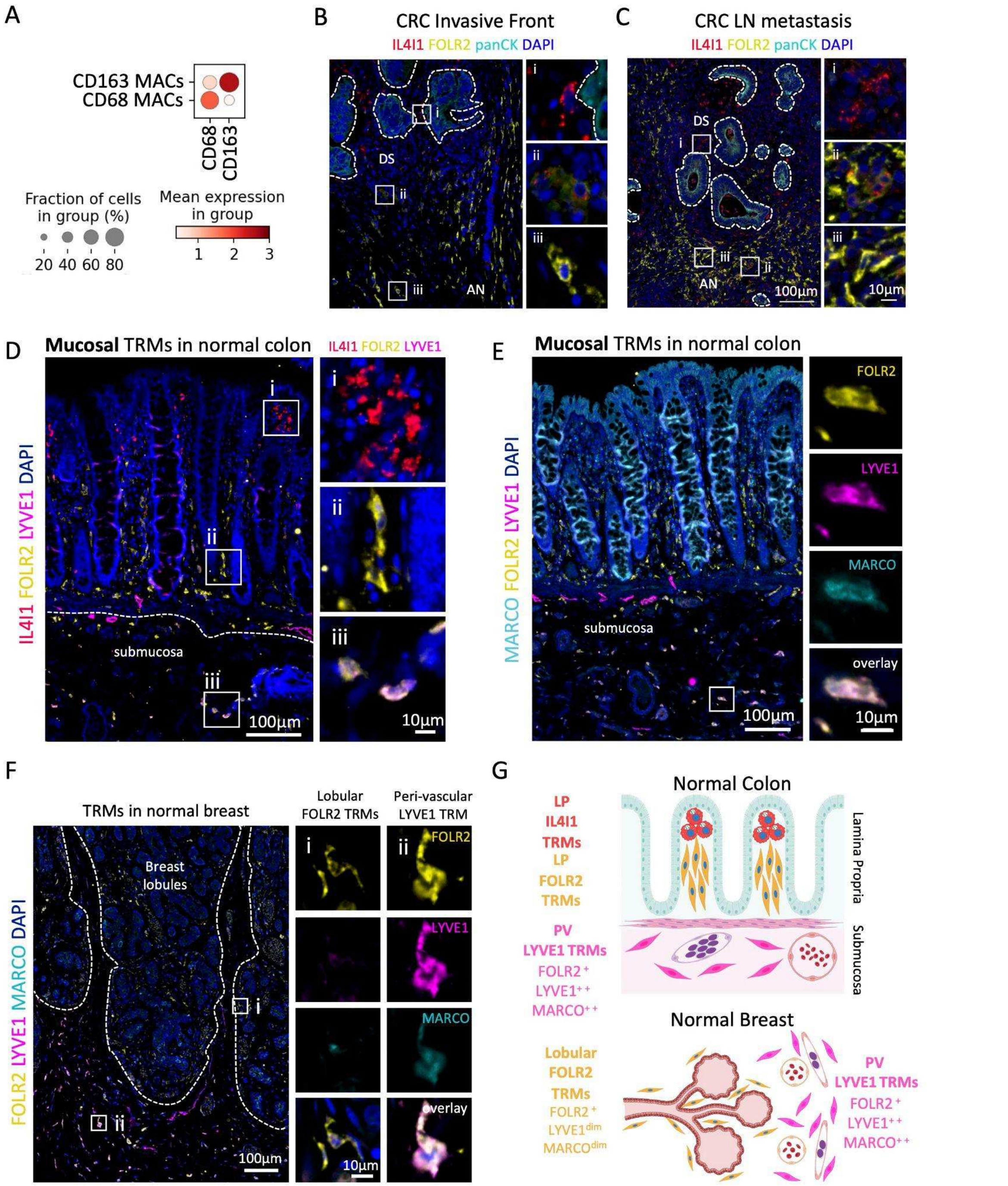

Fig S2. IL4I1, FOLR2, LYVE1, and MARCO label spatially segregated TRM niches in normal Colon and Breast, related to Fig 2 (**A**) Average protein expression in CD68 Macs and CD163 Macs. (**B-C**) Immunofluorescence (IF) images show IL4I1, FOLR2, and panCK signal distribution in (**B**) invasive front of CRC, and (**C**) CRC Lymph Node (LN) metastasis. DS- desmoplastic stroma, AN- adjacent normal. (**D**) IF images show 3 TRM layers marked by IL4I1, FOLR2, and LYVE1 in normal colon mucosa and submucosa. Note that LYVE1 also stains normal lymph vessels. (**E**) IF image shows that FOLR2^{+}, LYVE1^{+} TRMs in normal colon submucosa are MARCO^{+}. (**F**) IF images show TRMs in normal breast marked by FOLR2, LYVE1, and MARCO, depending on whether they are Lobular (i) or Peri-vascular (ii). (**G**) The schematic shows the distribution of TRM populations in normal colon mucosa and submucosa (*top*) and around normal breast glands (*bottom*). (**B**,**C**,**D**,**E,F**) Close-up images on the right correspond to boxed regions on the left. The scale bar of 10 µm is identical for all close-up images.

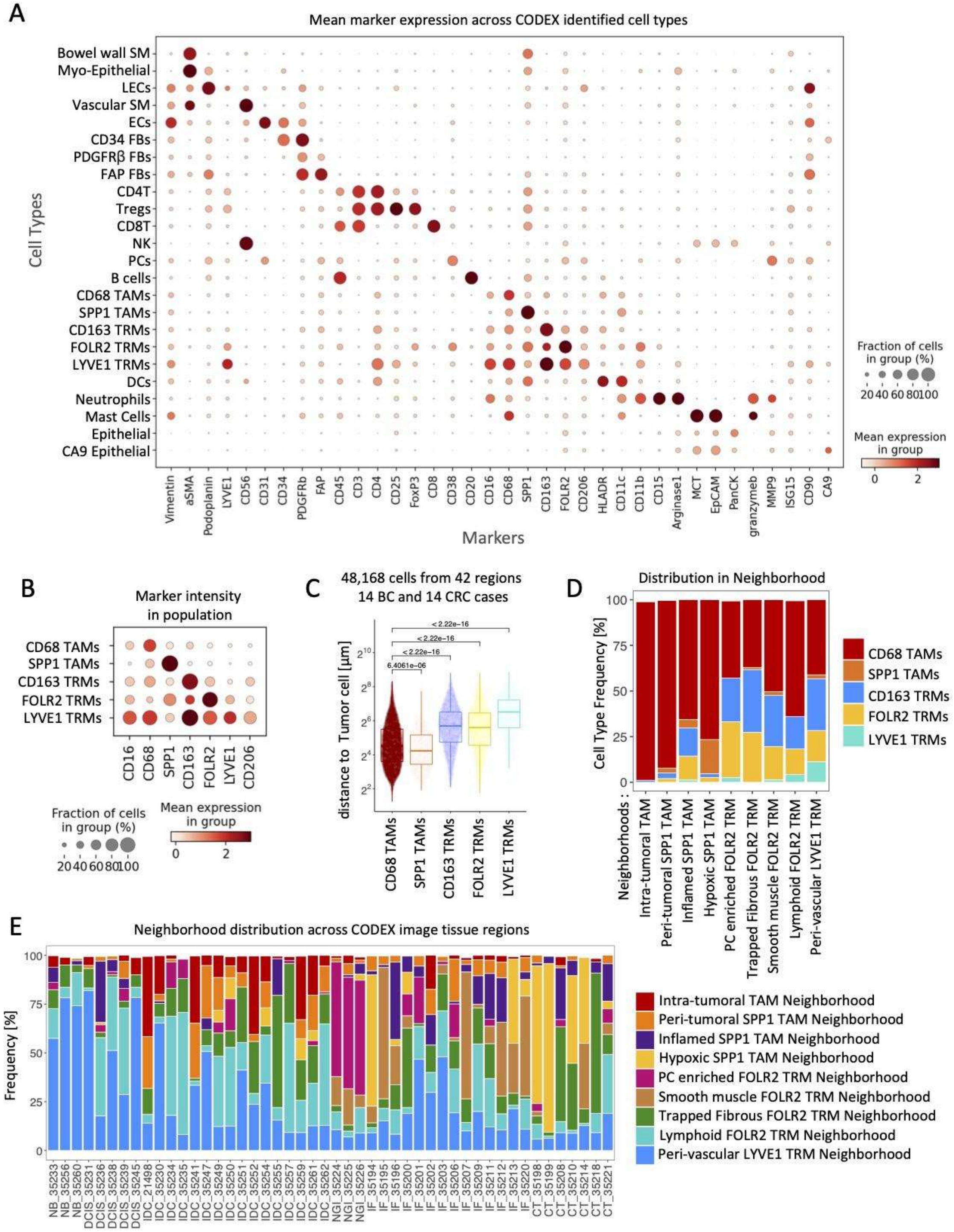

Fig S3. CODEX multichannel imaging reveals cellular interactions in macrophage niches, related to Fig 4
(**A**) Dotplot shows average normalized CODEX marker intensity per identified cell type. (**B**) Dotplot shows average normalized CODEX marker intensity per identified macrophage population. (**C**) Distance (µm) of CD68 TAMs, SPP1 TAMs, CD163 TRMs, FOLR2 TAMs and LYVE1 TRMs to the closest tumor cell. Cells were identified on CODEX images. P values were calculated with a linear mixed-effect model with Bonferroni's corrections for multiple comparisons. (**D**) Barplot shows the distribution of CODEX macrophage populations across macrophage neighborhoods. (**E**) Barplot shows the distribution of macrophage neighborhoods across CODEX imaged tissue regions.

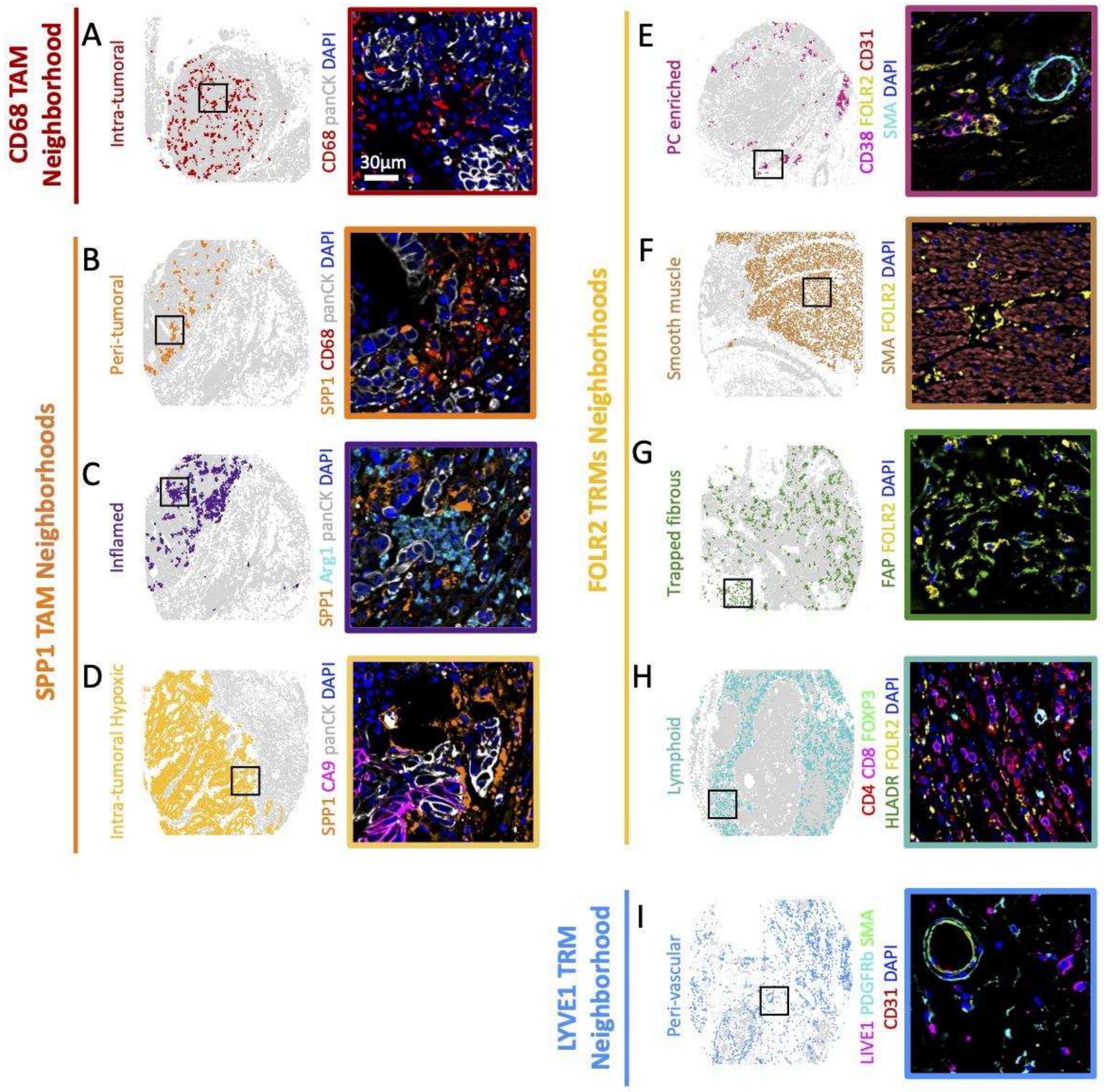

Fig S4. CODEX macrophage neighborhoods, related to Fig 4

(**A-I**) Representative *Left*: neighborhood distribution dotplots and *Right*: CODEX images showing cell types enriched in discussed CODEX macrophage neighborhoods. Close-up images on the right correspond to boxed regions on the left. Scale bar of 10 µm is identical for all close-up images. Panels B-D, F-G show CRC areas, panels A,E,H-I show BC areas.

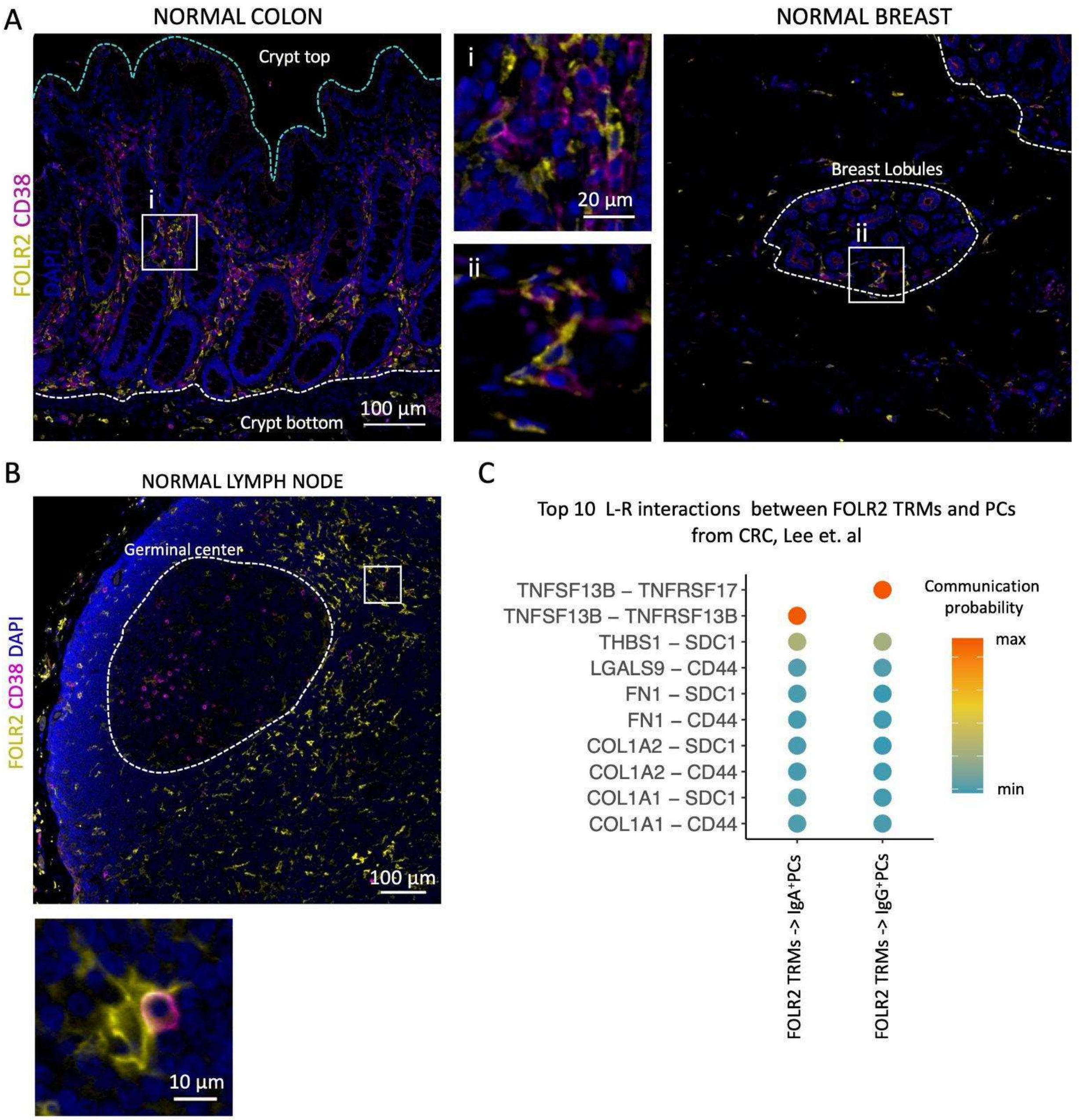

Fig S5. FOLR2 TRMs spatially colocalize with plasma cells and may maintain long-lived plasma cell tissue niche, related to Fig 5

(**A**) Immunofluorescence (IF) images show spatial interaction of FOLR2 TRMs with CD38 PCs in *Left:* the middle and bottom of the colon lamina propria and *Right:* normal breast gland. Middle: Close-up images in the middle correspond to boxed regions on the top and bottom IF images. The scale bar of 20 µm is identical for both close-up images. (**B**) IF images show PCs marked by CD38 and FOLR2 TRMs marked by FOLR2 in the normal lymph node. Close-up image on the bottom corresponds to boxed regions on the top. (**C**) Dotplot shows top 10 Ligand and Receptor interactions with the highest communication probability between IgA+PCs or IgG+PCs and FOLR2 TRMs in CRC scRNA Seq dataset of Lee et. al.

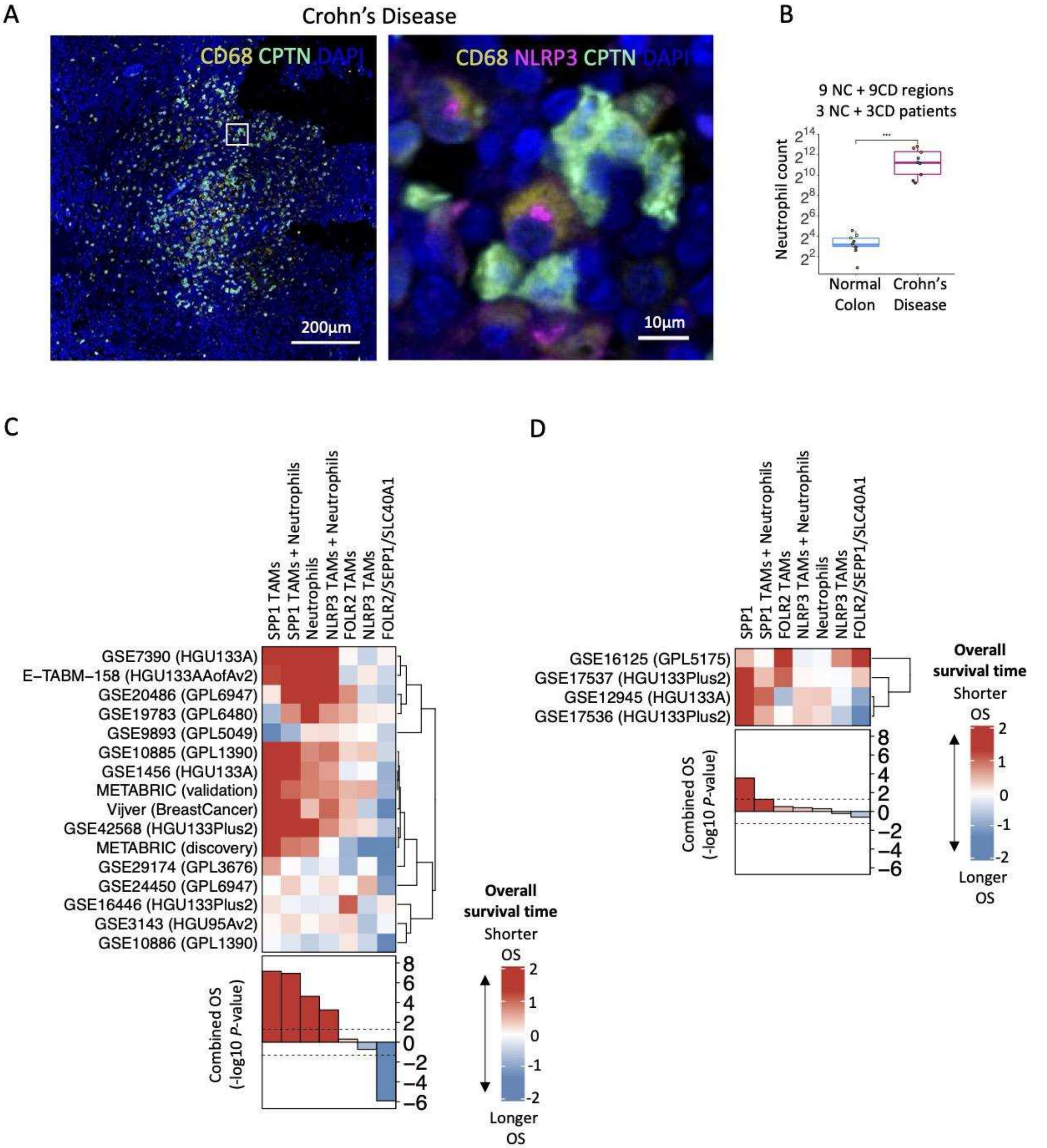

Fig S6. NLRP3 inflammasome activation is spatially associated with neutrophil infiltration in Crohn's Disease, related to Fig 6

(**A**) *Left:* IF images show CD68, CPTN and DAPI staining of a representative region of macrophage infiltrate in Crohn's Disease. *Right:* Close-up image on the right corresponds to boxed region on the left IF image and shows NLRP3, CD68, CPTN and DAPI staining. (**B**) Quantification of the number of neutrophils present in 9 normal colon submucosa and 9 macrophage infiltrated Crohn's Disease areas with NLRP3 specks. *P* value was computed using a two-sided Wilcoxon's rank-sum test. (**C**) Overall survival associations across 16 BC datasets. (**D**) Overall survival associations across 4 CRC datasets.

Supplementary Table 1. List of IHC and IF antibodies

Antibody	Clone	Vendor	Cat#	RRID	conc
Calprotectin	MAC387	Abcam	ab22506	AB_447111	1:1000
CD163	D6U1J	Cell Signaling	93498	AB_2800204	1:200
CD68 mouse	KP1	BioLegend	916104	AB_2616797	1:800
CD68 rabbit	D4B9C	Cell Signaling	76437	AB_2799882	1:200
CD68-555	KP1	Abcam	ab279323	AB_307338	1:50
FOLR2	OTI4G6	Novus	NBP2-45693	AB_2723188	1:100
IL4I1	EPR22070	Abcam	ab222102		1:200
LYVE1	AF2089	R&D	AF2089	AB_35514	1:50
MARCO	Polyclonal	Novus	NBP2-39004		1:100
NLRP3	Polyclonal	Sigma	ABF23		1:4000
panCK-AF647	AE-1/AE-3	Novus	NBP2-33200AF647	AB_963125	1:200
SPP1	HPA027541	Millipore Sigma	HPA027541-100UL	AB_10601446	1:500
Donkey anti-Goat IgG (H+L) Highly Cross-Adsorbed Secondary Antibody, Alexa Fluor Plus 647	Polyclonal	Thermo Fisher Scientific	A32849	AB_2762840	1:100
Donkey anti-Mouse IgG (H+L) Highly Cross-Adsorbed Secondary Antibody, Alexa Fluor Plus 555	Polyclonal	Thermo Fisher Scientific	A32773	AB_2762848	1:100
Donkey anti-Rabbit IgG (H+L) Highly Cross-Adsorbed Secondary Antibody, Alexa Fluor Plus 488	Polyclonal	Thermo Fisher Scientific	A32790	AB_2762833	1:100
Goat anti-Mouse IgG (H+L) Highly Cross-Adsorbed Secondary Antibody, Alexa Fluor Plus 555	Polyclonal	Thermo Fisher Scientific	A32727	AB_2633276	1:100

Goat anti-Mouse IgG (H+L) Highly Cross-Adsorbed Secondary Antibody, Alexa Fluor Plus 647	Polyclonal	Thermo Fisher Scientific	A32728	AB_2633277	1:100
Goat anti-Mouse IgG (H+L) Highly Cross-Adsorbed Secondary Antibody, Alexa Fluor™ Plus 488	Polyclonal	Thermo Fisher Scientific	A32723	AB_2633275	1:100
Goat anti-Rabbit IgG (H+L) Cross-Adsorbed Secondary Antibody, Alexa Fluor™ 488	Polyclonal	Thermo Fisher Scientific	A-11008	AB_143165	1:100
Goat anti-Rabbit IgG (H+L) Highly Cross-Adsorbed Secondary Antibody, Alexa Fluor Plus 555	Polyclonal	Thermo Fisher Scientific	A32732	AB_2633281	1:100
Goat anti-Rabbit IgG (H+L) Highly Cross-Adsorbed Secondary Antibody, Alexa Fluor Plus 647	Polyclonal	Thermo Fisher Scientific	A32733	AB_2633282	1:100

Supplementary Table 2. List of CODEX antibodies

Antibody	Clone	Vendor	Cat#	RRID
Arginase-1	polyclonal	Novus	NBP1-32731	RRID:AB_10003985
aSMA	polyclonal	Abcam	ab5694	RRID:AB_2223021
CA9	polyclonal	R&D	AF2188	RRID:AB_416562
CD11b	EPR1344	abcam	ab216445	RRID:AB_2864378
CD11c	EP1347Y	AbCam	ab216655	RRID:AB_2864379
CD15	MMA	BD	559045	RRID:AB_397181
CD16	D1N9L	Cell signaling	24326S	RRID:AB_2798877
CD163	EDHu-1	Novus	NB110-40686	RRID:AB_714951
CD20	rIGEL/773	Novus	NBP2-54591	RRID:AB_2864380
CD206	Polyclonal	R&D	AF2534	RRID:AB_2063019
CD25	4C9	Cell Marque	custom	RRID:AB_1157926

CD3	MRQ-39	Cell Marque	custom	RRID:AB_2864399
CD31	C31.3 + C31.7 + C31.10	Novus Bio	NBP2-47785	RRID:AB_2864381
CD34	QBEnd/10 + HPCA1/764	Novus	NBP2-47909-0.1mg	RRID:AB_2864382
CD38	EPR4106	abcam	ab176886	RRID:AB_2864383
CD4	EPR6855	Abcam	ab181724	RRID:AB_2864377
CD45	2B11 + PD7/26	Novus	NBP2-34528	RRID:AB_2864384
CD56	MRQ-42	Cell Marque	custom	RRID:AB_2861293
CD68	KP-1	Biolegend	916104	RRID:AB_2616797
CD8	C8/144B	Cell Marque	custom	RRID:AB_2864400
CD90	EPR3132	abcam	ab221607	RRID:AB_10563647
EpCAM	D9S3P	Cell signaling	14452	RRID:AB_2736866
FAP	Polyclonal	R&D	AF3715	RRID:AB_2102369
FOLR2-Biotin	OTI4G6	Novus	NBP2-70763B	RRID:AB_2723188
FoxP3	236A/E7	Invitrogen	14-4777-80	RRID:AB_467555
granzyme-B	EPR20129-217	Abcam	ab219803	RRID:AB_2910576
HLADR	EPR3692	AbCam	ab215985	RRID:AB_2864390
ISG15	polyclonal	Thermo Fisher	15981-1-AP	RRID:AB_2126302
LYVE1	AF2089	R&D	AF2089	RRID:AB_35514
Mast cell tryptase	AA1	Abcam	ab2378	RRID:AB_303023
MMP9	L51/82	Biolegend	819701	RRID:AB_2564833
pan-CK	C-11	Biolegend	628602	RRID:AB_439775
PDGFRb	Y92	Abcam	ab215978	RRID:AB_2894841
Podoplanin	D2-40	Biolegend	916606	RRID:AB_2565820
SPP1	HPA027541	Millipore Sigma	HPA027541-100UL	RRID:AB_10601446

Vimentin	RV202	BD	550513	RRID:AB_393716

Supplementary Table 3. CODEX runs metadata

Colon array CODEX cycle record

		DAPI		A488			Cy3			Cy5			
Well #	Cycle #			antibody	oligo	exposure time	antibody	oligo	exposure time	antibody	oligo	exposure time	Cycle #
A12	1	Hoechst	10	blank		500	blank		500	blank		500	1
B1	2	Hoechst	10	Vimentin	62	200	CD4	76	500	CD16	26	500	2
B2	3	Hoechst	10	aSMA	69	133	LYVE1	46	500	FoxP3	20	500	3
B3	4	Hoechst	10	CD15	14	117.647	PDGFRb	44	500	CD56	29	333	4
B4	5	Hoechst	10	pan-CK	67	50	CD34	38	333	granzyme-B	3	200	5
B5	6	Hoechst	10	blank		1.6	CD90	51	333	CD11c	49	333	6
B6	7	Hoechst	10	blank		1.6	Podoplanin	32	250	CD45	56	250	7
B7	8	Hoechst	10	blank		1.6	Arginase-1	43	250	CD11b	28	333	8
B8	9	Hoechst	10	blank		1.6	CD31	68	200	CD8	8	166.67	9
B9	10	Hoechst	10	blank		500	blank		500	blank		500	10
B10	11	Hoechst	10	blank		1.6	EpCAM	59	200	CD38	66	333	11
B11	12	Hoechst	10	blank		1.6	ISG15	42	200	HLADR	65	117.647	12
B12	13	Hoechst	10	blank		1.6	CD25	24	166	CD68	70	83	13
C1	14	Hoechst	10	blank		1.6	MMP9	80	166	CA9	53	66.67	14
C2	15	Hoechst	10	blank		1.6	CD163	45	166	CD20	48	83.33	15
C3	16	Hoechst	10	blank		1.6	FAP	79	166	SPP1	5	83	16
C4	17	Hoechst	10	blank		1.6	CD3	77	133	Mast cell tryptase	59	20	17
C5	18	Hoechst	10	blank		1.6	CD206	55	133	blank		1.6	18
C7	19	Hoechst	10	blank		1.6	FOLR2	SA-PE (2.5uL)	500	DRAQ5		166	19

Breast array CODEX cycle record

		DAPI		A488			Cy3			Cy5			
Well #	Cycle #			antibody	oligo	exposure time	antibody	oligo	exposure time	antibody	oligo	exposure time	Cycle #
A12	1	Hoechst	10	blank		500	blank		500	blank		500	1
B1	2	Hoechst	10	Vimentin	62	200	CD4	76	500	CD16	26	500	2
B2	3	Hoechst	10	aSMA	69	133	LYVE1	46	500	FoxP3	20	500	3
B3	4	Hoechst	10	CD15	14	117.647	PDGFRb	44	500	CD56	29	333.33	4
B4	5	Hoechst	10	pan-CK	67	50	CD34	38	333	granzyme-B	3	200	5
B5	6	Hoechst	10	blank		1.6	CD90	51	333	CD11c	49	333.33	6
B6	7	Hoechst	10	blank		1.6	CD38	66	333	CD45	56	250	7
B7	8	Hoechst	10	blank		1.6	Arginase-1	43	250	CD11b	28	166	8
B8	9	Hoechst	10	blank		1.6	CD31	68	200	CD8	8	166.67	9
B9	10	Hoechst	10	blank		500	blank		500	blank		500	10
B10	11	Hoechst	10	blank		1.6	EpCAM	59	200	SPP1	5	133	11
B11	12	Hoechst	10	blank		1.6	ISG15	42	200	HLADR	65	117.647	12
B12	13	Hoechst	10	blank		1.6	CD25	24	166	CD68	70	83	13
C1	14	Hoechst	10	blank		1.6	MMP9	80	166	CA9	53	66.67	14
C2	15	Hoechst	10	blank		1.6	CD163	45	166	CD20	48	83.33	15
C3	16	Hoechst	10	blank		1.6	FAP	79	166	Mast cell tryptase	59	20	16
C4	17	Hoechst	10	blank		1.6	Podoplanin	32	166	blank		1.6	17
C5	18	Hoechst	10	blank		1.6	CD3	77	133	blank		1.6	18
C6	19	Hoechst	10	blank		1.6	CD206	55	83	blank		1.6	19
C7	20	Hoechst	10	blank		1.6	FOLR2	SA-PE (2.5uL)	500	DRAQ5		500	20

Supplementary Table 4. Overview of the cohort of clinically-annotated bulk tumor transcriptomes.

Dataset ID	GEO, ArrayExpres platform	Platform name	Tumor type	No. analyzed samples	PMID	First author
METABRIC (validation)	GPL10558	Illumina HumanHT-12 V4.0 expression beadchip	Breast cancer	984	27161491, 22522925	Pereira, Curtis
METABRIC (discovery)	GPL10558	Illumina HumanHT-12 V4.0 expression beadchip	Breast cancer	979	27161491, 22522925	Pereira, Curtis
van de Vijver	NA	Hu25K microarrays	Breast cancer	295	12490681	van de Vijver
GSE24450	GPL6947	Illumina HumanHT-12 V3.0 expression beadchip	Breast cancer	183	22171747, 21542898, 22102859	Heikkinen, Muranen, Peurala
GSE1456	GPL96	Affymetrix Human Genome U133A Array	Breast cancer	159	16280042, 16813654	Pawitan, Hall

GSE3143	GPL8300	Affymetrix Human Genome U95 Version 2 Array	Breast cancer	158	16273092	Bild
GSE7390	GPL96	Affymetrix Human Genome U133A Array	Breast cancer	155	17545524, 25788628	Desmedt, Patil
GSE9893	GPL5049	MLRG Human 21K V12.0	Breast cancer	155	18347175	Chanrion
GSE10886	GPL1390	Agilent Human 1A Oligo UNC custom Microarrays	Breast cancer	149	19204204	Parker
E-TABM-158	A-AFFY-76	Affymetrix High Throughput Array U133AA of Av2	Breast cancer	129	17157792	Chin
GSE19783	GPL6480	Agilent-014850 Whole Human Genome Microarray 4x44K G4112F (Probe Name version)	Breast cancer	110	21364938, 26321095, 23382830	Enerly, Haakensen, Aure
GSE16446	GPL570	Affymetrix Human Genome U133 Plus 2.0 Array	Breast cancer	107	21422418, 20098429, 20189874, 26484051	Desmedt, Li, Juul, Haibe-Kains
GSE42568	GPL570	Affymetrix Human Genome U133 Plus 2.0 Array	Breast cancer	104	23740839	Clarke
GSE20486	GPL6947	Illumina HumanHT-12 V3.0 expression beadchip	Breast cancer	97	20551037, 24662924	Parris, Parris
GSE29174	GPL3676	NKI-CMF Homo sapiens 35k oligo array	Breast cancer	96	21586611	Farazi
GSE10885	GPL1390	Agilent Human 1A Oligo UNC custom Microarrays	Breast cancer	45	19435916	Hennessy
GSE17536	GPL570	Affymetrix Human Genome U133 Plus 2.0 Array	Colorectal cancer	177	19914252, 22115830, 25916654, 30606770	Smith, Freeman, Williams, Chen
GSE12945	GPL96	Affymetrix Human Genome U133A Array	Colorectal cancer	62	19399471	Staub
GSE17537	GPL570	Affymetrix Human Genome U133 Plus 2.0 Array	Colorectal cancer	55	19914252, 22115830, 25916654, 30606770	Smith, Freeman, Williams, Chen
GSE16125	GPL5175	Affymetrix Human Exon 1.0 ST Array [transcript (gene) version]	Colorectal cancer	32	19672874	Reid

Supplementary Table 5. List of genes used for outcome predictions

FOLR2 TAMs	NLRP3 TAMs	NLRP3 TAMs + Neutrophils	SPP1 TAMs	SPP1 TAMs + Neutrophils	LYVE1 TRMs
FOLR2	NLRP3	NLRP3	SPP1	SPP1	FOLR2
CXCR4	IL1B	IL1B	MMP12	MMP12	SEPP1
CD163	CXCL1	CXCL1	MMP9	MMP9	SLC40A1
SELENOP	CXCL2	CXCL2	INHBA	INHBA	
C1QA	CXCL8	CXCL8	KLK6	KLK6	
MS4A7	INHBA	INHBA	S100A14	S100A14	
	PLAUR	PLAUR	KLK10	KLK10	
		S100A8	PLAUR	PLAUR	
		S100A9	CTSL	CTSL	
		CSF3R	FABP5	FABP5	
		MPZ		S100A8	
		HCAR3		S100A9	
		SERPINB2		CSF3R	
		CXCL8		MPZ	
		CD300E		HCAR3	
				SERPINB2	
				CXCL8	
				CD300E	
